# Neuroanatomical Alterations in the CNTNAP2 Mouse Model of Autism Spectrum Disorder

**DOI:** 10.3390/brainsci13060891

**Published:** 2023-05-31

**Authors:** Tanya Gandhi, Cade R. Canepa, Tolulope T. Adeyelu, Philip A. Adeniyi, Charles C. Lee

**Affiliations:** Department of Comparative Biomedical Sciences, School of Veterinary Medicine, Louisiana State University, Baton Rouge, LA 70806, USA

**Keywords:** autism, CNTNAP2, corticothalamic, perineuronal nets, parvalbumin, interneurons, cerebral cortex

## Abstract

Autism spectrum disorder (ASD) is associated with neurodevelopmental alterations, including atypical forebrain cellular organization. Mutations in several ASD-related genes often result in cerebral cortical anomalies, such as the abnormal developmental migration of excitatory pyramidal cells and the malformation of inhibitory neuronal circuitry. Notably here, mutations in the CNTNAP2 gene result in ectopic superficial cortical neurons stalled in lower cortical layers and alterations to the balance of cortical excitation and inhibition. However, the broader circuit-level implications of these findings have not been previously investigated. Therefore, we assessed whether ectopic cortical neurons in CNTNAP2 mutant mice form aberrant connections with higher-order thalamic nuclei, potentially accounting for some autistic behaviors, such as repetitive and hyperactive behaviors. Furthermore, we assessed whether the development of parvalbumin-positive (PV) cortical interneurons and their specialized matrix support structures, called perineuronal nets (PNNs), were altered in these mutant mice. We found alterations in both ectopic neuronal connectivity and in the development of PNNs, PV neurons and PNNs enwrapping PV neurons in various sensory cortical regions and at different postnatal ages in the CNTNAP2 mutant mice, which likely lead to some of the cortical excitation/inhibition (E/I) imbalance associated with ASD. These findings suggest neuroanatomical alterations in cortical regions that underlie the emergence of ASD-related behaviors in this mouse model of the disorder.

## 1. Introduction

Autism spectrum disorder (ASD) comprises a range of behavioral conditions arising from the atypical development of the nervous system. Advances in ASD research, global autism awareness, international health policies and improvements in diagnostic criteria have led to the present estimates for the prevalence of autism, which is currently around 65 per 10,000 cases worldwide [1,2,3] and 1 in 54 cases in the US [4,5,6,7]. Individuals with ASD manifest social, communication challenges and restricted, repetitive behaviors [8,9,10,11]. These core behavioral symptoms are often associated with comorbidities such as hyperactivity, anxiety, epilepsy and aggression [12,13]. There is a lack of effective therapeutic interventions directed at the core behavioral symptoms of ASD [7,14]. Presently, behavioral therapies and pharmacological interventions target specific behavioral deficits and/or associated comorbidities based on the individual need [15,16]. However, novel treatment interventions are needed that treat the mechanisms underlying these core autistic behaviors.

Several genetic and environmental risk factors contribute to the development of ASD and converge on similar neural mechanisms [17,18,19]. Genetic mutations account for a considerable part of autism risk [20,21], with a higher concordance for the development of the autistic phenotype in monozygotic twins (92%) than in dizygotic twins (10%) [15,22]. Several genes have been identified that play an important role in the etiological heterogeneity of ASD, and affect many processes during neural development, including establishing appropriate neural connectivity, excitatory/inhibitory (E/I) balance and neurotransmitter signaling.

Among the risk genes associated with autism, mutations in the contactin-associated protein-like 2 (CNTNAP2) gene leads to a syndromic form of ASD and cortical dysplasia focal epilepsy (CDFE) [23,24]. Common genetic variants of CNTNAP2 are associated with increased susceptibility to autism [23,25]. The CNTNAP2 gene encodes a neural transmembrane scaffolding protein CASPR2 that plays an important role in neural development, such as clustering of voltage-gated potassium channels at the juxtaparanodal region of myelinated axons, dendritic spine formation, neuron-glia interaction, and cortical laminar organization [26,27,28,29].

The CNTNAP2 knockout mouse model recapitulates several core behavioral features of ASD, such as impairments in social interaction and vocal communication, repetitive and restricted behaviors, and associated comorbid symptoms including hyperactivity and seizures [30]. Mutant mice also demonstrate defects in cortical neuronal migration, reduced cortical interneuron number, abnormal cortical network activity, and ectopic neurons in the corpus callosum [30], and corresponds with MRI brain scans and histological analysis of resected brain tissues from human patients with CDFE syndrome [26]. In addition, CNTNAP2 expression is enriched in cortical circuits which are important for language development [31], is implicated in frontal lobe abnormal functional connectivity [32] and leads to altered brain structural connectivity [33]. Hence, CNTNAP2 is required for normal neural development, with mutations leading to alterations in the structure and connectivity of the brain regions implicated in ASD behaviors.

In particular, the cerebral cortex and thalamus are implicated in ASD [34,35,36,37,38,39,40]. The thalamus plays an essential role in controlling information flow among cortical areas. In addition to receiving a modulatory (feedback) input from layer 6 of the cortex, the higher-order thalamic nuclei also receive a driver (feedforward) input from layer 5 of the cortex. Hence, the higher-order thalamic nuclei relay information between cortical areas by input from layer 5 of one cortical area to the thalamorecipient layer of another area. This represents a potential alternate cortico-thalamo-cortical route for information flow [41,42,43]. Hence, elucidating alterations in the feedforward “driver” thalamocortical circuit contribution to the etiology of ASD will provide new insight into the neuroanatomical alterations associated with the mutation of the CNTNAP2 gene.

As alluded to above, the cerebral cortex has a laminar organization in which excitatory and inhibitory cortical neurons are arranged in six distinct layers [44]. Several ASD mouse models show abnormal neuronal migration of superficial cortical layer neurons to lower cortical layers during development, including the CNTNAP2 model [30]. This is similar to the ectopic cortical migration patterns observed in humans with ASD [45,46]. These ectopic projections might result in altered information flow through the cortico-thalamo-cortical pathway that might be involved in sensorimotor function impairment and the emergence of autistic behaviors [47,48,49]. Hence, exploring these corticothalamic circuit alterations will provide an understanding of aberrant neuroanatomical connections and mechanisms underlying the emergence of autistic behaviors.

In addition, the regulation of these cortical output pathways depends on an intricate balance of excitatory and inhibitory (E/I) circuits [40,50,51,52]. Cortical E/I balance is mediated by finely tuned interactions among excitatory glutamatergic pyramidal neurons and inhibitory GABAergic interneurons. Both glutamatergic and GABAergic pathways are crucial during neural development, by facilitating cortical organization via migration and the positioning of excitatory pyramidal cells and inhibitory interneurons [53]. Alterations in cortical inhibitory circuitry and signaling is associated with autism and many other brain disorders [54,55,56,57,58,59,60,61,62,63,64]. Fast-spiking interneurons expressing parvalbumin (PV) are an important sub-population of inhibitory GABAergic cells [65,66]. The fast-spiking nature of PV-positive cells imposes high metabolic demands that render them sensitive to oxidative stress and damage [67,68]. Interestingly, extracellular matrix structures, such as perineuronal nets (PNNs) assemble at the end of critical periods, protecting the PV cells against oxidative damage and stabilizing their activity [69,70,71,72,73,74]. In addition to protecting interneurons from oxidative stress, PNNs also play an important role in synaptic regulation and in the modulation of plasticity via controlling the onset and closure of critical periods of development [75,76]. Therefore, alterations to PNN structure and function affects the PV cells inhibitory activity and consequently E/I balance.

Therefore, in this study, we assessed whether ectopic neurons in lower layers of primary sensory cortical areas in CNTNAP2^−/−^ mice formed aberrant connections with related thalamic structures, by using a retrograde tract tracing strategy. Furthermore, we assessed whether the development of PNNs and PVs were dysregulated in sensory cortical regions of CNTNAP2^−/−^ mice, potentially resulting in the E/I imbalance and aberrant cortical activity underlying autistic behaviors.

## 2. Materials and Methods

### 2.1. Animal Care and Housing

Wild-type C57BL/6J (strain: 000664) and mutant CNTNAP2 (strain: 017482) breeder pairs were obtained from the Jackson Laboratory (Bar Harbor, ME, USA) and the offspring were used in the study. All experiments were approved by the Institutional Animal Care and Use Committee (IACUC) of the Louisiana State University (Baton Rouge, LA, USA), approval code: IACUCAM-20-012. Mice were housed in a temperature and humidity-controlled room with a 12 h light/dark cycle with lights on at 7:00 a.m. and food and water provided ad libitum.

### 2.2. Genotyping

Tails snips of wild-type C57BL/6J and CNTNAP2 knockout (KO) breeder mice were collected for genotyping. Genotyping was conducted using the PCR of mouse tail DNA (Mouse Genotype, Escondido, CA, USA). The primers used were wild-type reverse 5′ACA CCA GGG GCA AGA ATT G3′, mutant reverse 5′CGC TTC CTC GTG CTT TAC GGT AT3′ and common primer 5′CTG CCA GCC CAG AAC TGG3′ (Jackson Laboratory, Bar Harbor, ME, USA). The wild-type allele band was 389bp in size and the mutant allele band was 350 bp in size.

### 2.3. Retrograde Tract-Tracing

Adult mice were used to investigate the corticothalamic and corticocortical connectivity of ectopic layer 5 neurons in primary sensory cortical regions. The mice were anesthetized with intraperitoneal injections of a ketamine (100 mg/kg body weight) and xylazine (20 mg/kg body weight) cocktail. Toe-pinch withdrawal reflex confirmed the loss of sensation in the animals. General use eyedrops were used for lubricant and protection purposes. The head of the animal was then placed in a stereotaxic apparatus (Stoelting, Wood Dale, IL, USA). The scalp was cleaned using 70% alcohol, a midline incision was made to expose the scalp, and a dental drill was used to perform a craniotomy above the injection site. Injections of tracer were performed as described below. Subsequently, the scalp incision site was cleaned and sutured. After suturing, a generic triple antibiotic cream was applied over the sutured skin for preventing any infections after the surgery. Further, buprenorphine was given intraperitoneally (0.1 mg/kg concentration) to manage the pain after craniotomy and during the recovery period. During the recovery period of 7–10 days, animals were observed daily for any manifestation of distress and infection, up to the time of sacrifice.

To study the layer 5 corticothalamic projections, non-primary sensory thalamic nuclei were targeted for stereotaxic injections of the retrograde tracer. The corresponding coordinates of non-primary sensory thalamic nuclei were determined using the stereotaxic atlas and the AP and ML positions were marked relative to bregma [63]. A 500 μL Neuros Syringe (Hamilton, Reno, NV, USA) was used to stereotaxically inject 4% retrograde tracer hydroxystilbamidine (Fluoro-GoldTM) (#80023, Biotium, CA, USA) into the injection site. A 0.1 μL of solution was injected at a rate of 0.04 μL/min using a micro syringe pump (World Precision Instruments, Sarasota, FL, USA). Injections were targeted bilaterally to posterior medial thalamic nucleus (POM) [anterior-posterior (AP) −2.06 mm; lateral (ML) ±1.25; ventral (V) −3.15], dorsal division of the medial geniculate thalamic nucleus (MGNd) (AP −3.28 mm; ML ±2 mm; V −3 mm), lateral posterior thalamic nucleus or pulvinar (LP/PUL) (AP −2.11 mm; ML ±1.73 mm; V −2.73 mm) and mediodorsal thalamic nucleus (MD) (AP −1.06 mm; ML ±0.35 mm; V −3 mm). The needle was gradually inserted to and extracted from the injection site over 5 min. The animals were allowed to recover for a period of 7–8 days to allow adequate expression and transport of the retrograde tracer.

### 2.4. Histology

Mice were anesthetized via isoflurane inhalation. Anesthetized mice were then transcardially perfused with 1× phosphate-buffered saline (PBS) (concentration 10 mM, pH 7.4), followed by perfusion with 4% paraformaldehyde solution (PFA) diluted in 1× PBS from 32% stock solution (#15714, Electron Microscopy Sciences, Hatfield, PA, USA). The whole brain samples were harvested and postfixed in 4% PFA solution overnight at 4 °C. Samples were stored next day in 4% PFA solution containing 30% sucrose for cryopreservation at 4 °C until cryosectioning. Brain samples were cryosectioned on a freezing cryostat coronally at 40 μm thickness and collected in 24 well plates containing 1× PBS solution (pH 7.4) and stored at 4 °C until immunostaining. Free-floating sections containing the sensory cortical regions (auditory, visual, and somatosensory) were separately stained for CUX-1 and for PNN and PV expressions.

To examine cortical lamination, sections stained for CUX-1 expression were rinsed three times (5 min each) in 1× PBS (10 mM) solution and blocked using normal goat serum (NGS) (#S-1000 Vector Laboratories, Burlingame, CA, USA) and 0.03% Triton-X 100 solution prepared in 1× PBS (10mM) for 1 h at room temperature. After blocking, sections were incubated overnight at 4 °C in primary antibody solution, i.e., CUX-1 rabbit polyclonal antibody (#11733-1-AP, Proteintech, IL, USA) at a dilution of 1:500 in blocking solution. Sections were washed three times (5 min each) in 1× PBS (10 mM) before incubation with secondary goat anti-rabbit Alexa Fluor 568 conjugated antibody (#ab175695, Abcam, Boston, MA, USA) at a dilution of 1:500 for 1 h at room temperature. Next, sections were rinsed three times (5 min each) in 1× PBS solution. Following rinsing, sections were mounted on gelatin coated slides and cover-slipped using Vectashield anti-fade mounting media (#H-1400, Vector Laboratories, Burlingame, CA, USA).

Separate sections were stained for PNN and PV expression [77]. After rinsing sections three times (5 min each) in 1× PBS (10mM), sections were incubated in Avidin D and biotin solutions for 15 min each to block biotin and streptavidin binding sites (#SP-2002, Vector Laboratories, Burlingame, CA, USA). Next, sections were rinsed three times (5 min each) in 1× PBS and then incubated with biotinylated WFA/WFL (Wisteria floribunda agglutinin/lectin) (#B-1355, Vector Laboratories, Burlingame, CA, USA) solution at 19.8 μg/mL (dilution 1:500) in 1× PBS (10mM) overnight at room temperature. Afterwards, sections were rinsed again in 1× PBS three times (5 min each) before incubation with streptavidin conjugated with Alexa Fluor 568 (CF-29035, Biotium, CA, USA) at a dilution of 1:500 in 1× PBS solution for 1 h at room temperature. For staining of PV-positive neurons, sections were washed in 1× PBS three times (5 min each) and blocked using normal goat serum (NGS) (#S-1000 Vector Laboratories, Burlingame, CA, USA) and 0.03% Triton X-100 for 1 h at room temperature. Next, sections were incubated overnight at 4 °C in primary antibody solution, i.e., rabbit polyclonal anti-parvalbumin antibody (#ab11427, Abcam, Boston, MA, USA) at a dilution of 1:500 in blocking solution. Sections were rinsed again before incubation with secondary goat anti-rabbit Alexa Fluor 488 antibody (#ab150081, Abcam, Boston, MA, USA) at a dilution of 1:500 for 1 h at room temperature. After final rinsing in 1× PBS, sections were mounted on gelatin slides and cover-slipped using Vectashield anti-fade mounting media (#H-1400, Vector Laboratories, Burlingame, CA, USA).

### 2.5. Imaging and Quantification

**Figure 1:**
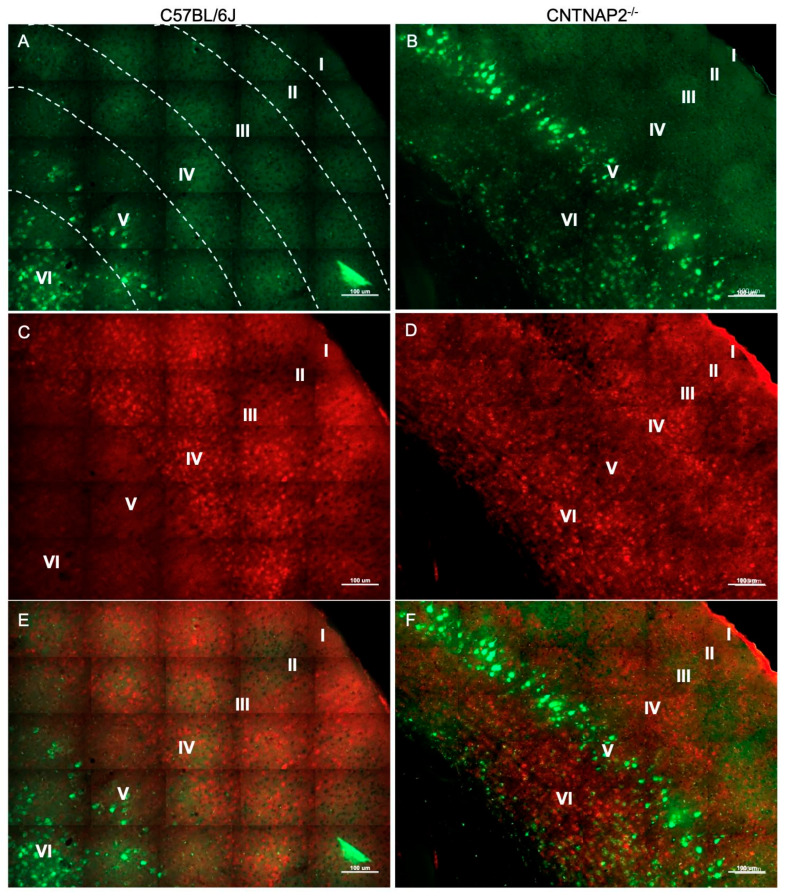
Primary somatosensory (S1) corticothalamic connectivity in wild-type (**A**,**C**,**E**) and CNTNAP2^−/−^ (**B**,**D**,**F**) mice. Images of (**A**,**B**) fluorogold-labeled cells (green); (**C**,**D**) CUX-1 positive cells (red); and (**E**,**F**) co-localized cells in somatosensory cortex of C57BL/6J and CNTNAP2^−/−^ mice. Roman numerical notation I–VI represents cortical layers 1–6 (separated by dashed line). Scale bar 100 μm.

Fluorescent images were obtained using a Zeiss Observer Z1 microscope (Carl Zeiss Microscopy, White Plains, NY, USA) and Nanozoomer whole slide scanner (Hamamatsu Photonics, Bridgewater, NJ, USA). Image data was viewed, analyzed, and quantified using Axiovision Zeiss microscope software (Carl Zeiss Microscopy, NY, USA), Image J (NIH, Bethesda, MD, USA) and NDP2 software (Ver 2.9.29, 2023, Hamamatsu Photonics, Bridgewater, NJ, USA). Photomicrographs obtained from the Zeiss Observer Z1 were automatically photomontaged from the motorized stage scans, resulting in a montaging artifact in the figures constructed from those scans (e.g., Figure 1). CUX-1 positive, retrogradely labeled and co-localized cells were counted in a blinded and randomized way to the observed scanned image data. A standard atlas was utilized to identify the sensory cortical regions (A1, S1 and V1) of interest (ROIs) based on cytoarchitecture and gross morphology [63]. Cell counting was conducted manually by an investigator in a blinded manner across defined ROIs. CUX-1 positive cells, retrogradely labeled, and CUX-1 co-localized with retrogradely labeled cells were counted separately in the determined area over the ROIs. An average of 3–4 ROIs were quantified per sample. Data analyses were performed using GraphPad software (Ver. 9.5, 2023, San Diego, CA, USA). Unpaired student’s *t*-tests were used to determine statistical difference in the stereological data. Threshold for significance was set at *p* < 0.05. Data were expressed as mean ± standard error of mean (SEM) and significance were presented as *, 0.05 > *p* > 0.01; **, 0.01 > *p* > 0.001; ***, 0.001 > *p* > 0.0001.

## 3. Results

### 3.1. Genotyping

We validated the genetic alterations in the CNTNAP2^−/−^ mice compared to the C57BL/6J wild-type background animals, by genotyping from tail snips of both mutant and background strains. The mutant band PCR product is around 350 bp, and the wild-type band size is around 389 bp. (Figure S1: line A1 and B1 represent homozygous mutant bands and C1 is wild-type band).

### 3.2. Ectopic Cortical Neurons Project to Sensory Thalamic Nuclei in CNTNAP2 KO Mice

**Figure 2:**
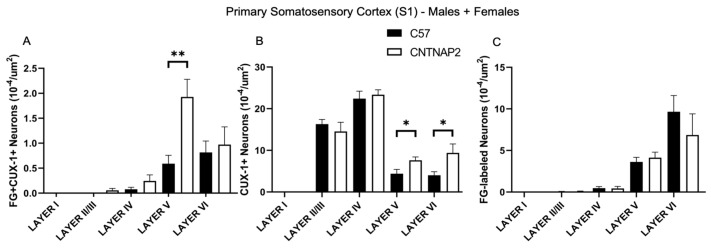
Quantification of ectopic layer V neurons in primary somatosensory cortex (S1) forming aberrant connections with non-primary posterior medial thalamic nucleus (POM) in CNTNAP2^−/−^ mice compared to C57BL/6 mice. (**A**) Density of co-localized fluorogold-labeled and CUX-1 positive cells; (**B**) CUX-1 positive cells only; (**C**) fluorogold-labeled cells only in combined male and female mice. C57 (*n* = 9) and CNTNAP2^−/−^ (*n* = 8) mice. Data expressed as mean ± SEM (*p* < 0.05) (*p* < 0.1). Significance were presented as *, 0.05 > *p* > 0.01; **, 0.01 > *p* > 0.001.

**Figure 3:**
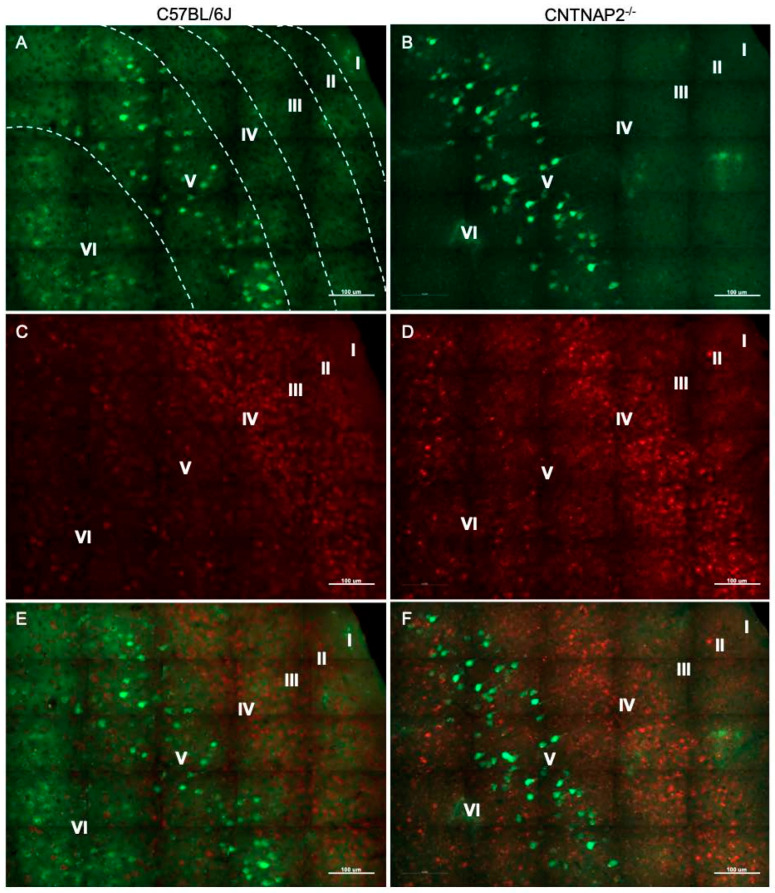
Primary auditory (A1) corticothalamic connectivity in wild-type (**A**,**C**,**E**) and CNTNAP2^−/−^ (**B**,**D**,**F**) mice. Immunofluorescence images of (**A**,**B**) fluorogold-labeled cells (green); (**C**,**D**) CUX-1 positive cells (red); and (**E**,**F**) co-localized cells in primary auditory cortex of C57BL/6J and CNTNAP2^−/−^ mice. Roman numerical notation I–VI represents cortical layers 1–6 (separated by dashed line). Scale bar 100 μm.

**Figure 4:**
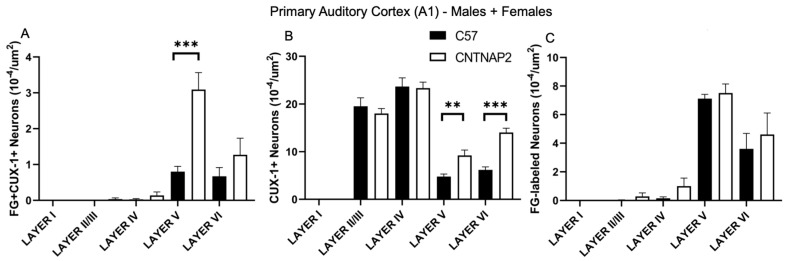
Quantification of ectopic layer 5 neurons in primary auditory cortex (A1) forming aberrant connections with non-primary dorsal medial geniculate nucleus (DMGN) in CNTNAP2^−/−^ mice compared to C57BL/6 mice. (**A**) Density of co-localized fluorogold-labeled and CUX-1 positive cells; (**B**) CUX-1 positive cells only; (**C**) fluorogold-labeled cells only in combined male and female mice. C57 (*n* = 7) and CNTNAP2^−/−^ (*n* = 6) mice. Data expressed as mean ± SEM (*p* < 0.05). Significance were presented as **, 0.01 > *p* > 0.001; ***, 0.001 > *p* > 0.0001.

**Figure 5:**
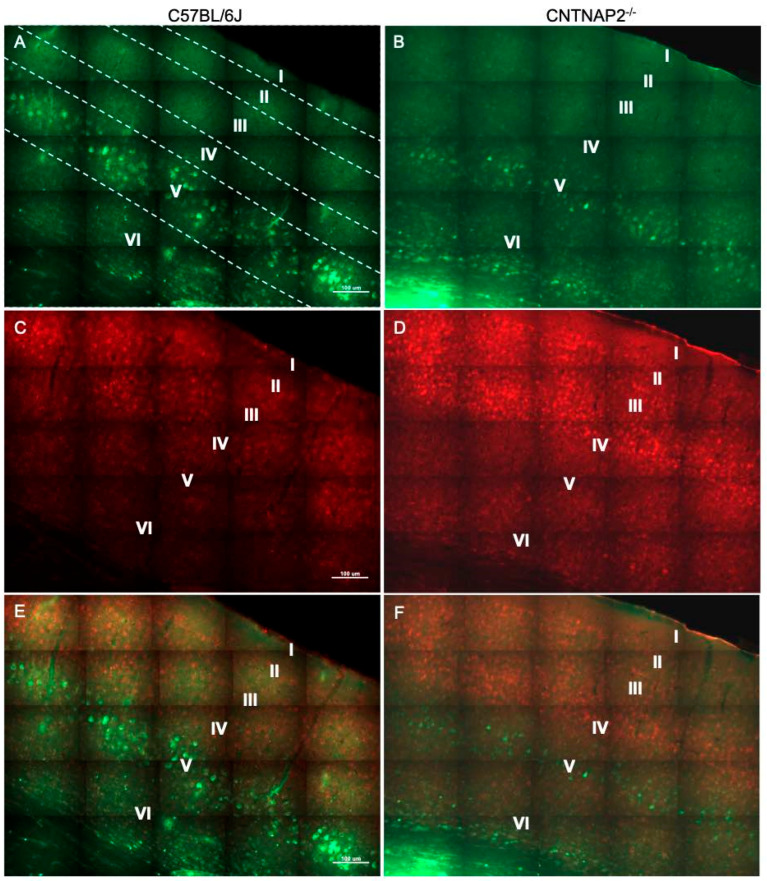
Primary visual (V1) corticothalamic connectivity in wild-type (**A**,**C**,**E**) and CNTNAP2^−/−^ (**B**,**D**,**F**) mice. Immunofluorescence images of (**A**,**B**) fluorogold-labeled cells (green); (**C**,**D**) CUX-1 positive cells (red); and (**E**,**F**) co-localized cells in primary visual cortex of C57BL/6J and CNTNAP2^−/−^ mice. Roman numerical notation I–VI represents cortical layers 1–6 (separated by dashed line). Scale bar 100 μm.

**Figure 6:**
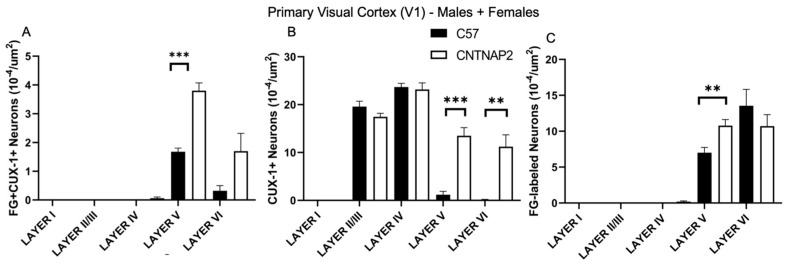
Quantification of ectopic layer 5 neurons in primary visual cortex (V1) forming aberrant connections with non-primary lateral posterior nucleus/pulvinar (LP/PUL) in CNTNAP2^−/−^ mice compared to C57BL/6 mice. (**A**) Density of co-localized fluorogold-labeled and CUX-1 positive cells; (**B**) CUX-1 positive cells only; (**C**) fluorogold-labeled cells only in combined male and female mice. C57 (*n* = 6) and CNTNAP2^−/−^ (*n* = 6) mice. Data expressed as mean ± SEM (*p* < 0.05) (*p* < 0.1). Significance were presented as **, 0.01 > *p* > 0.001; ***, 0.001 > *p* > 0.0001.

To assess connectivity of ectopic layer 5 neurons with higher-order sensory thalamic nuclei, we employed a retrograde tract tracing approach in CNTNAP2^−/−^ and C57BL/6J (C57) mice injected with retrograde tracer fluorogold (FG) in the non-primary thalamic nuclei, i.e., posterior medial nucleus (PoM), dorsal division of the medial geniculate body (MGBd) and lateral posterior nucleus/pulvinar (LP/PUL) ( Figures S2 and S3). Following thalamic injections, retrograde labeling was found in lower layers 5 and 6 of the corresponding primary sensory cortical areas, i.e., primary somatosensory (S1), primary auditory (A1) and primary visual (V1) regions (Figures S2, S3, S10–S16, S19, S22, S25, S28 and S31). Laminar organization of ectopic cortical neurons was determined with antibodies to CUX-1, which is a marker for neurons normally localized to the superficial cortical layers 2–3, with CUX1-labeling in lower layers indicative of ectopically localized neurons (Figures S10–S16, S19, S22, S25, S28 and S31) [30,64]. We assessed co-localized FG-labeled and CUX-1+ cells in the primary sensory cortical regions (Figure 1, Figure 2, Figure 3, Figure 4, Figure 5 and Figure 6).

CNTNAP2^−/−^ mice exhibited a significant increase in the distribution of CUX-1+ cells in lower cortical layers 5 and 6 of the S1, A1 and V1 regions (Figure 2, Figure 4 and Figure 6). The number of double-labeled cells for retrograde tracer (FG) and CUX-1+ in lower layer 5 of the S1, A1 and V1 regions are significantly different between control and KO mice (Figure 2, Figure 4 and Figure 6). These findings confirmed the presence of abnormally migrated neurons in lower layer 5 of the primary sensory cortical areas in the CNTNAP2^−/−^ mice as compared to the C57BL/6J. Furthermore, the novel finding of double-labeled cells indicates that these ectopic layer 5 neurons in the CNTNAP2^−/−^ mice are anatomically connected with the respective non-primary thalamic nuclei, potentially resulting in atypical information flow through the cortico-thalamo-cortical pathway and likely contributing to sensorimotor impairments and repetitive behaviors in CNTNAP2 mutant mice.

As assessed by gender, some differences in altered connectivity of the ectopic layer 5 neurons between control and KO mice were observed. Interestingly, the female, but not male, CNTNAP2^−/−^ group showed a significant increase in the CUX-1+ and double-labeled cell density in the lower layer 5 of the S1 region as compared to their gender-match C57BL/6J group (Figures S4 and S5). There is no difference in the density of the retrogradely labeled cells in the lower layer 5 in either the male or female CNTNAP2^−/−^ and WT groups (Figures S5 and S7). Interestingly, the double-labeled cells were significantly different in the male and female CNTNAP2^−/−^ groups in lower layer 5 of the S1, A1 and V1 regions than the WT group (Figures S4, S6 and S8). Further, both the male and female CNTNAP2^−/−^ groups showed increases in CUX-1+ and double-labeled cell density in the lower layer 5 of the A1 and V1 regions as compared to the C57BL/6J group (Figures S6–S9). Intriguingly, there was a decrease in the CUX-1+ cell density in the upper layer 2/3 in the male CNTNAP2^−/−^ group and in the middle layer 4 in the female CNTNAP2^−/−^ group in the V1 region compared to the C57BL/6J group (Figure S9). There was no difference in the density of the retrogradely labeled cells in the lower layer 5 of the S1 and A1 regions in either the male or female CNTNAP2^−/−^ and C57BL/6J groups (Figures S5 and S7). However, the female CNTNAP2^−/−^ group exhibits increased density of retrogradely labeled cells in the lower layer 5 of the V1 region as compared to the C57BL/6J group (Figure S9). The variations in the CUX-1 positive, retrogradely labeled and double-labeled cell densities across different cortical regions between the control and KO male and the female groups could be attributed to the gender differences and/or variability in the diffusion of the retrograde label in the mice.

### 3.3. Perineuronal Nets (PNNs) and Parvalbumin (PV)-Positive Interneurons Exhibit Altered Development with Age in the Primary Sensory Cortical Regions (S1, A1, V1) of CNTNAP2 KO Mice

**Figure 7:**
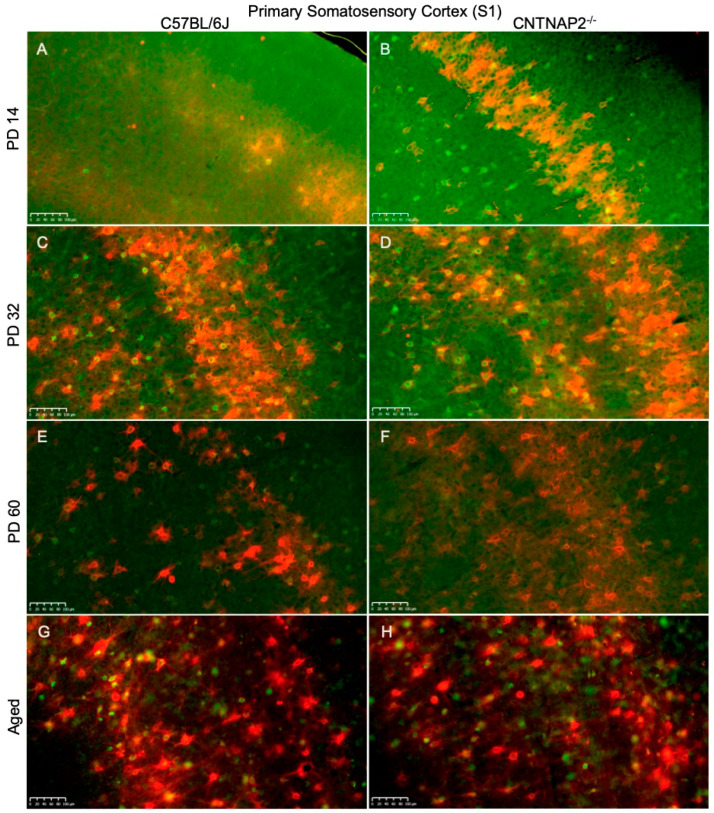
Primary somatosensory cortical (S1) distribution of co-localized PNNs (red) and PV+ cells (green) in C57BL/6J (**A**,**C**,**E**,**F**) and CNTNAP2^−/−^ (**B**,**D**,**F**,**H**) mice at (**A**,**B**) PD 14, (**C**,**D**) PD 32, (**E**,**F**) PD 60 and (**G**,**H**) aged. There is a developmental increase in PNNs, PV neurons and co-localized cells in CNTNAP2^−/−^ mice in the S1 region compared to control animals. Scale bars: 100 μm.

**Figure 8:**
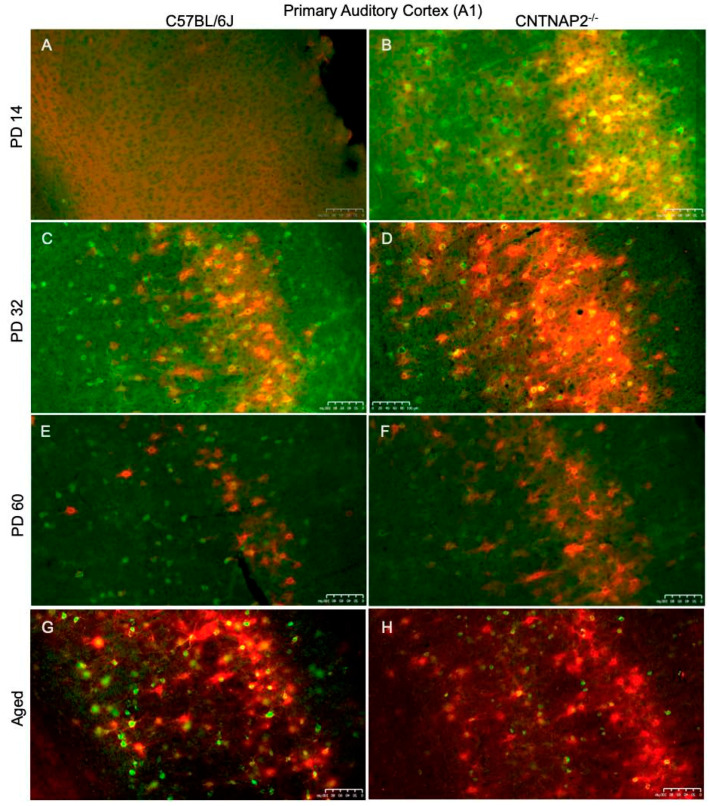
Primary auditory cortical (A1) expression of co-localized PNNs (red) and PV+ cells (green) in C57BL/6J (**A**,**C**,**E**,**F**) and CNTNAP2^−/−^ (**B**,**D**,**F**,**H**) mice at (**A**,**B**) PD 14; (**C**,**D**) PD 32; (**E**,**F**) PD 60; and (**G**,**H**) aged. PNNs, PV neurons and co-localized cells developmentally increase in CNTNAP2^−/−^ mice in the A1 region compared to control animals. Scale bars: 100 μm.

**Figure 9:**
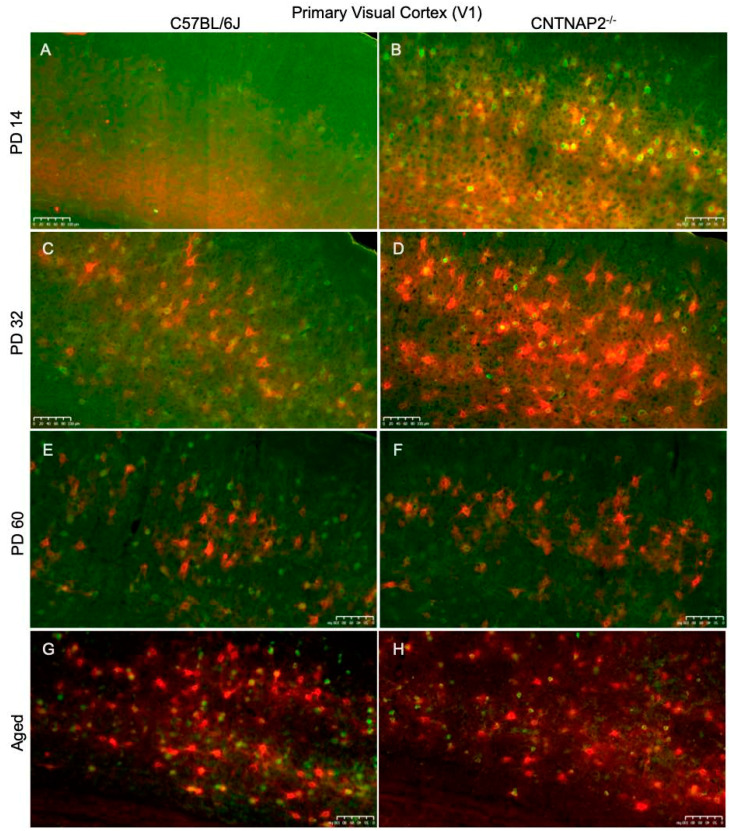
Primary visual cortical (V1) development of co-localized PNNs (red) and PV+ cells (green) in C57BL/6J (**A**,**C**,**E**,**F**) and CNTNAP2^−/−^ (**B**,**D**,**F**,**H**) mice at (**A**,**B**) PD 14; (**C**,**D**) PD 32; (**E**,**F**) PD 60; and (**G**,**H**) aged. The expression of PNNs, PV neurons and co-localized is greater in CNTNAP2^−/−^ mice in the V1 region compared to control animals. Scale bars: 100 μm.

**Figure 10:**
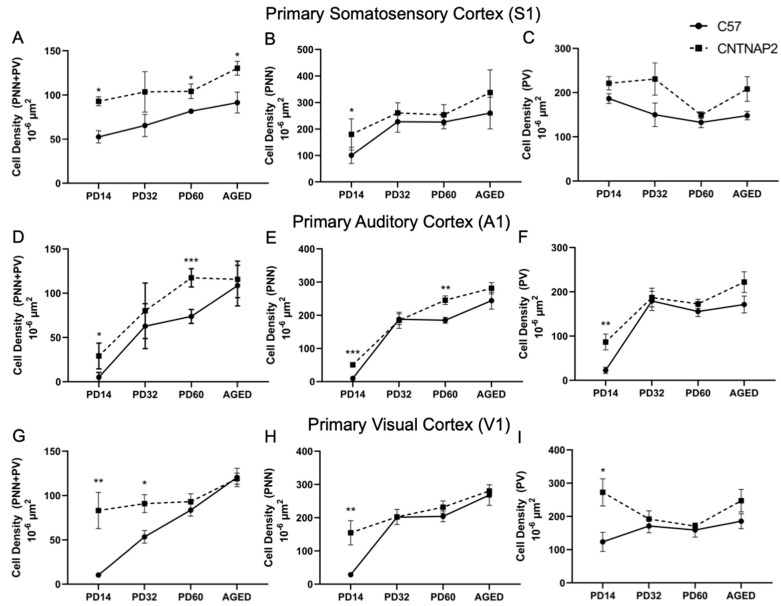
Quantitation of the developmental expression of PNNs (middle column), PV+ neurons (right column), and co-localized cells (left column). (**B**,**E**,**H**). (**A**–**C**) Primary somatosensory cortex (S1). (**D**–**F**) Primary auditory cortex (A1). (**G**–**I**) Primary visual cortex (V1) of C57BL/6J and CNTNAP2^−/−^ mice at PD 14, PD 32, PD 60 and aged. Data expressed as mean ± SEM (*p* < 0.05). Significance were presented as *, 0.05 > *p* > 0.01; **, 0.01 > *p* > 0.001; ***, 0.001 > *p* > 0.0001.

Perineuronal nets (PNNs), parvalbumin (PV)-positive interneurons and their co-localization in the primary sensory cortical areas (S1, A1, V1) were analyzed in brain sections of control C57BL/6J and CNTNAP2 mutant mice at different postnatal ages, i.e., PD 14 (neonates), PD 32 (young), PD 60 (adult) and PD395-PD425 (aged) (Figure 7, Figure 8 and Figure 9). The quantitative distribution of PNNs and PVs in different sensory cortical regions was determined from average counts in a defined ROI to obtain cell densities (see Methods) (Figure 10). At PD 14, the CNTNAP2^−/−^ group exhibited significantly increased densities of PNNs and their co-localization with PV+ cells in S1, A1 and V1 regions as compared to the control C57 group (Figure 7, Figure 8, Figure 9, Figure 10 and Figures S10–S12). Intriguingly, PV+ neuronal density was markedly elevated in the A1 and V1 regions in the CNTNAP2 mutant group in comparison to the C57 group at PD 14 (Figure 7, Figure 8, Figure 9, Figure 10 and Figures S10–S12). Unlike previous studies which reported a reduced PV+ cell number in the somatosensory cortex, striatum and hippocampus in CNTNAP2 KO mice at PD 14 [30], we observed no changes in the PV+ cell density in the primary somatosensory (S1) cortical region in the CNTNAP2 mutant mice at PD 14. We found abundant increases in PNNs and PVs in the S1, A1 and V1 regions as opposed to the control group at PD 14 (Figure 7, Figure 8, Figure 9, Figure 10 and Figures S10–S12).

By PD 32, the PNNs and PV+ neurons development continued in S1, A1 and V1 cortical regions in both the control and mutant groups (Figure 7, Figure 8, Figure 9 and Figures S13–S15). At this age, no statistical difference was observed between the groups in the PNNs, PVs and their co-localization densities in S1 and A1 regions, which was consistent with prior findings [78] (Figure 10). However, at PD 32, the CNTNAP2 mutant group exhibited a significant increase (70%) in PNNs co-localized with the PV-positive cells in the V1 region in contrast to the C57BL/6J group (Figure 7, Figure 8, Figure 9 and Figure 10). Prior studies have shown increased numbers of PNNs in the visual cortex at PD35 subsequently forming into fully developed PNNs at PD 70 [79,80]. In contrast to previous findings that reported a decrease in the PV+ cell number in the striatum and somatosensory cortex of the CNTNAP2^−/−^ mice at PD 14 and PD 30 [30,81], we found no difference in the PV+ cell density in the S1 region at PD 14 and PD 32 (Figure 10). However, we found an increase in the PV+ neuronal density in A1 and V1 of the CNTNAP2^−/−^ mice at PD 14, but not at PD 32 (Figure 10). At PD 32, around 40–45% of total PNNs in the defined unit area of the S1, A1 and V1 regions were co-localized with the PV+ cells in the CNTNAP2^−/−^ mice, whereas in the C57BL/6J mice, 27–33% of total PNNs were co-localized with the PVs in primary cortical areas (Figure 10).

At PD60, PNNs and PVs manifest fully developed shape and form with complex interlocking patterns (Figure 7, Figure 8, Figure 9, Figures S16, S19 and S22). Quantification of PNNs and their co-localization with PV+ neurons at PD 60 indicated a significant rise in their densities in the S1 and A1 regions in the CNTNAP2 mutant mice in comparison to the C57 control mice (Figure 10). CNTNAP2^−/−^ mice exhibit an approximately 28% and 59% increase in the co-localized cell densities compared to C57BL/6J mice in S1 and A1 regions, respectively (Figure 10). The CNTNAP2^−/−^ mice also exhibited increased PNN density in the A1 region at PD 60 (Figure 10 and Figure S19). However, no difference in densities of PNNs, PVs and their co-localization were observed between the groups in the V1 region at PD 60 (Figure 10 and Figure S22). Analysis by gender showed increased densities of PNNs and co-localized cells in the S1 and A1 regions in the male and female CNTNAP2^−/−^ groups at PD 60 (Figures S17, S18, S20 and S21). Previous observations found no reduction in the cortical PV+ levels in CNTNAP2^−/−^ mice at PD 70 [78], which is similar to our findings of PV+ cell density in S1, A1 and V1 regions in the CNTNAP2^−/−^ mice at PD 60 (Figure 10, Figures S17, S18, S20, S21, S23 and S24). Steady development and maturation of PNNs and PVs are observed in aged mice in both the genotypes (Figure 7, Figure 8, Figure 9, Figures S25, S28 and S31).

Aged CNTNAP2^−/−^ mice exhibited a significant increase in the co-localized cell density in the S1 region compared to the aged C57 mice (Figure 10). However, no significant differences were observed in the densities of PNNs, PVs and co-localized cells in the A1 and V1 regions in the aged CNTNAP2^−/−^ mice compared to the aged controls (Figure 10). Analysis by gender revealed that the aged female CNTNAP2^−/−^ mice showed a significant rise in the PNN density in the S1, A1 and V1 regions in contrast to the aged female C57BL/6J mice (Figures S27, S30 and S33). The aged female CNTNAP2^−/−^ mice also showed increased co-localized cell density in the S1 region (Figure S27). No significant differences were observed in the PNNs, PVs and co-localized cell densities in the aged male CNTNAP2^−/−^ mice in the S1, A1 and V1 regions compared to the aged controls (Figures S26, S29 and S32). Surprisingly, the aged female CNTNAP2^−/−^ group showed considerable elevation in the PV+ cell density in the A1 region compared to the aged female C57 group (Figure S30). These findings in the aged mice require further study with more sample size to understand if the trend towards higher PV-positive cell density in the primary cortical areas (S1, A1 and V1) in aged CNTNAP2^−/−^ mice is linked with changes in PV expression levels.

## 4. Discussion

**Figure 11:**
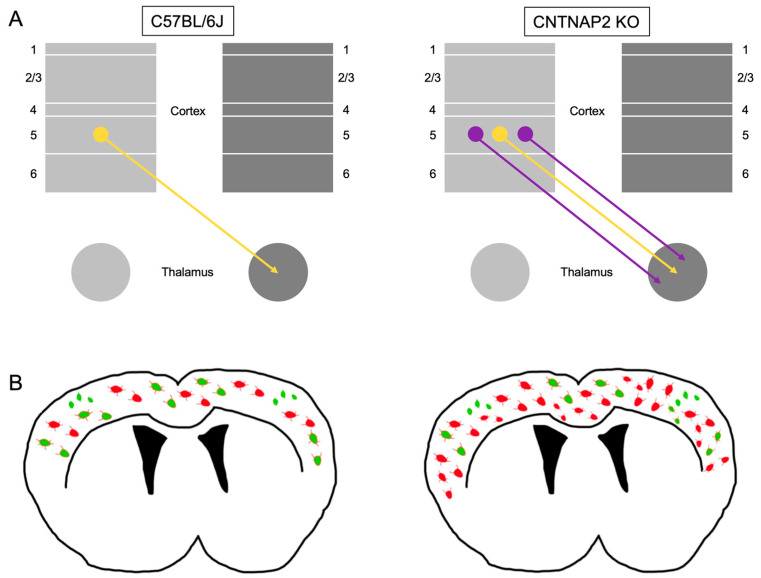
Schematic illustration of observed neuroanatomical alterations in the CNTNAP 2 mouse. (**A**) Illustration depicting ectopic neurons in lower cortical layer 5 (purple arrows) leading to enhanced corticothalamic connectivity in mutant CNTNAP2 mice compared to normal layer 5 connectivity observed in C57BL/6J animals (gold arrow). (**B**) Depiction of increase PNNs (red), PVs (green) and PNNs co-localized with PV neurons (red/green) in CNTNAP2 mice compared with C57BL/6J animals.

Genetic variability in ASD and the resultant underlying changes in connectivity and pathways leading to neurodevelopmental disruption are key factors in the pathogenesis of ASD [30,82,83,84,85,86,87,88]. Here, we found that the abnormal migration of neurons to cortical layer 5 results in altered corticothalamic connectivity in the CNTNAP2^−/−^ mice, perhaps leading to amplification of sensorimotor flow of information to thalamic and motor centers [42,89]. Such aberrant corticothalamic activity may account for the some of the observed repetitive and hyperactive behaviors observed in the CNTNAP2^−/−^ mice (Figure 11).

One consistent observation in ASD is the abnormal pattern of brain growth in regions crucial for social, communication and motor skill development [90,91,92]. For instance, social deficits in ASD are associated with impaired frontal brain functioning that mediates goal-directed reward activity and adaptive behaviors. Moreover, those with ASD may exhibit abnormal activation and synchronization patterns across various subcortical and cortical regions, including impaired functional connectivity in regions involved with language, social and cognitive tasks [93]. Neuropathological studies indicate cortical dysgenesis, with abnormalities in neuronal and cortical organization, such as disturbed cortical laminar patterns and increased numbers of neurons [94,95,96,97,98,99,100,101,102,103,104].

On a microcircuit level, cortical mini columns, which are anatomical and functional units consisting of vertical columns of neurons across cortical layers 2–6 of the brain, are abnormal in ASD. In autistic individuals, mini columns are smaller and more numerous, the cortex has disturbed lamination, and neuronal size is reduced with increased cell density compared to the control patients [94,105]. These observations suggest that cortical disorganization in ASD leads to altered functional connectivity and information processing in different areas of the brain, thereby affecting the development of behavior and communication in children [105,106,107,108].

In the present study, neuroanatomical analysis of CNTNAP2^−/−^ mice shows neuronal migration abnormalities in the sensory cortices and altered connectivity between the thalamus and cortex that are associated with autistic-like behaviors. Previous observations have shown cortical neuronal migration abnormalities in CNTNAP2^−/−^ mice [30] which is similar to aberrant cortical migration patterns observed in CDFE syndrome patients with the CNTNAP2 mutation [26]. Our results extend these prior findings by demonstrating that some of these ectopic cortical neurons form connections with the thalamus.

Cortical layer 5 plays an important role for the transfer of sensory information from primary cortical areas to non-primary thalamic nuclei, which conveys information further to higher sensory cortical regions [42,43,47,109,110]. These layer 5 projections serve as feedforward projections that drive information between cortical areas via an alternate route of information flow, i.e., the cortico-thalamo-cortical route in addition to the traditional direct corticocortical pathway from upper cortical layers 2 and 3 [41,42,47,48,49,89,111,112]. In addition, these corticothalamic neurons in layer 5 send projections to motor centers, thereby affecting generated motor behaviors [89]. Our findings suggest that the ectopic layer 5 corticothalamic connectivity likely contributes to aberrant sensorimotor activity via the alternate corticothalamic route. 

In addition, we found alterations in perineuronal nets (PNNs), parvalbumin-positive interneurons (PVs) and PNNs enwrapping PV-positive neurons in primary sensory cortical regions in CNTNAP2 mutant mice at different postnatal ages (Figure 11). Analysis by gender for PD 60 and aged mutant and control groups showed some variations in the densities of PNNs, PVs and co-localized cells between the male and female groups. These variations observed between the genders could be attributed to the gender differences and/or variability in the staining and observed background. These alterations suggest accelerated and erratic growth and maturation of PNNs and enwrapped PV cells that may be involved in the disruption of E/I balance leading to altered information processing, thereby contributing towards the abnormal behaviors observed in CNTNAP2 mutant mice. 

PV-positive interneurons regulate the output of excitatory principal neurons, development and synchronization of neural networks, resulting in normal information processing and cognitive flexibility [65,66,113,114]. Alterations in PV cell activity during the critical period of development might lead to abnormal neurophysiological effects and associated behavior symptoms [78,115,116]. At the end of the critical period, PV-positive neurons mature in parallel with the formation of extracellular matrix structures, the perineuronal nets around them. Perineuronal nets enwrap the PV-positive cells, resulting in the maintenance and stabilization of inhibitory network activity, modulating excitation and inhibition balance, plasticity and learning [69,70,71,117,118,119,120].

## 5. Conclusions

The neuroanatomical factors underlying the emergence of autism spectrum disorders are numerous and intricately linked. Our present findings demonstrate that both altered corticothalamic connectivity and expression of PNNs and GABAergic PV-positive interneurons in the primary sensory cortical regions are present in the CNTNAP2 KO mouse model of autism. These findings suggest that these alterations to corticothalamic circuits, PNNs and enwrapped PVs are among the neurobiological substrates for ASD-like behaviors. Thus, future work will require elaborating the network effects arising from these altered local and long-range circuits in ASD and related neurodevelopmental disorders.

## Data Availability

The data presented in this study are available on request from the corresponding author.

## Supplementary Materials

The following supporting information can be downloaded at: https://www.mdpi.com/article/10.3390/brainsci13060891/s1, Supplementary Figures S1–S33.
