# Supplementary material for: Neuroanatomical Alterations in the CNTNAP2 Mouse Model of Autism Spectrum Disorder
Source: Brain Sci. 2023 May 31;13(6):891. doi: 10.3390/brainsci13060891 (PMC10296611; doi:10.3390/brainsci13060891)
Supplement: Supplementary file 1 [file brainsci-13-00891-s001.zip › brainsci-2404980-supplementary.pdf]

Supplemental Information

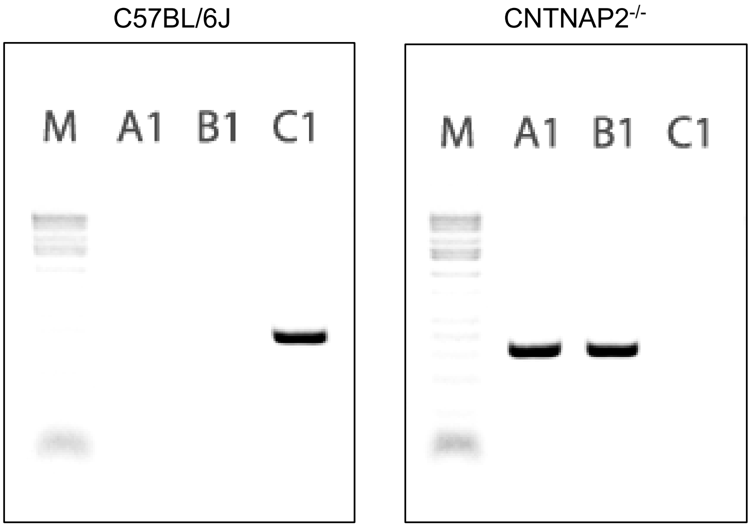

Figure S1. Agarose-gel images showing wildtype (C57BL/6J) and mutant (CNTNAP2$^{-/-}$) bands.

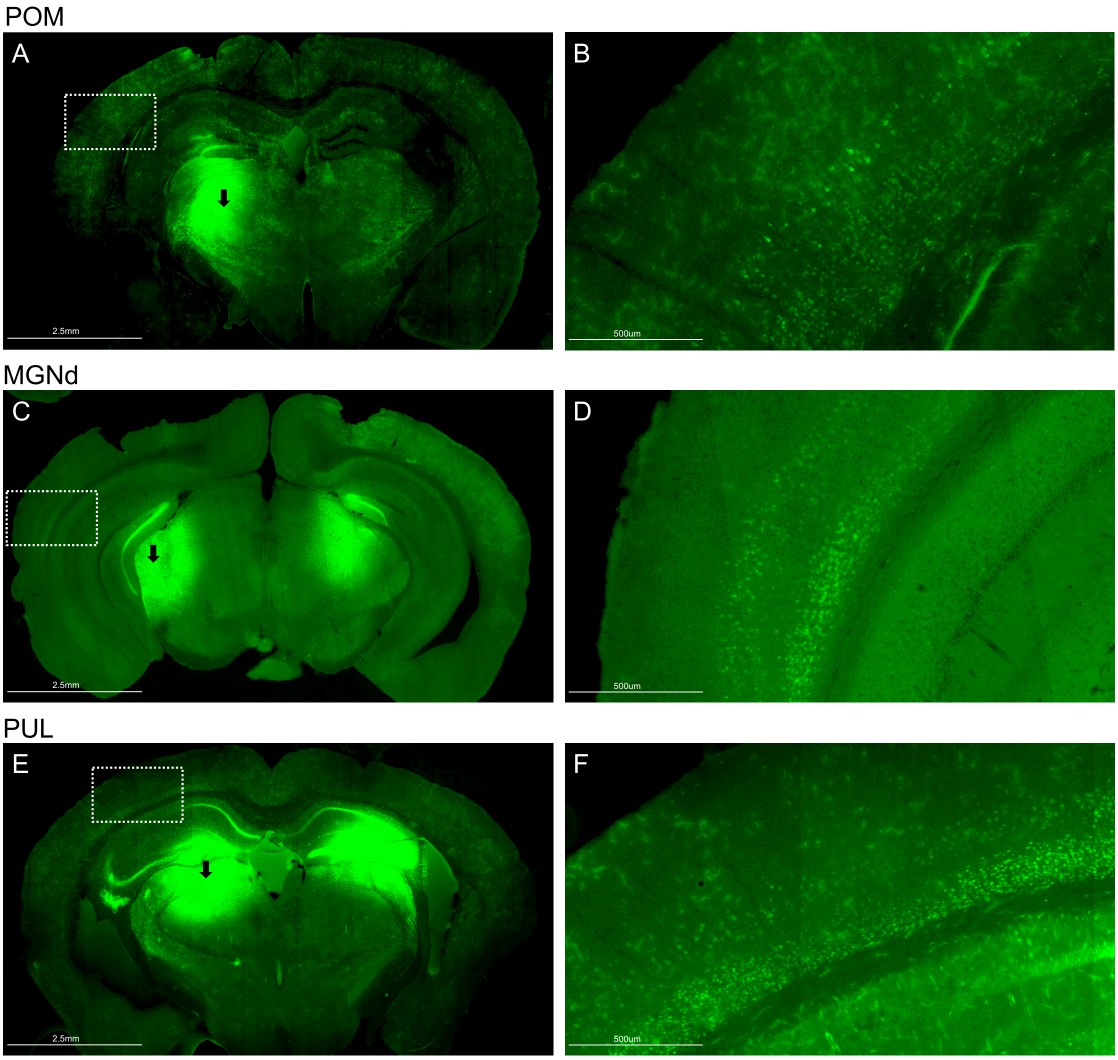

Figure S2. Representative images depicting neuroanatomical tract tracing injections and labeling in C57BL/6J mice of retrograde tracer injected in non-primary sensory thalamic nuclei. (A) posterior medial nucleus (POM) (indicated with arrow) with (B) retrogradely labeled cells in S1(enlarged image indicated by dashed line in A), (C) dorsal division of medial geniculate nucleus (MGNd) (indicated with arrow) with (D) retrogradely labeled cells in A1 (enlarged image indicated by dashed line in C), and (E) lateral posterior thalamic nucleus/pulvinar (PUL) (indicated with arrow) with (F) retrogradely labeled cells in V1 (enlarged image indicated by dashed line in E). Images A and B were horizontally flipped to match orientation with other images. Images A, C and E scale bar 2.5mm. Images B, D and F scale bar 500µm.

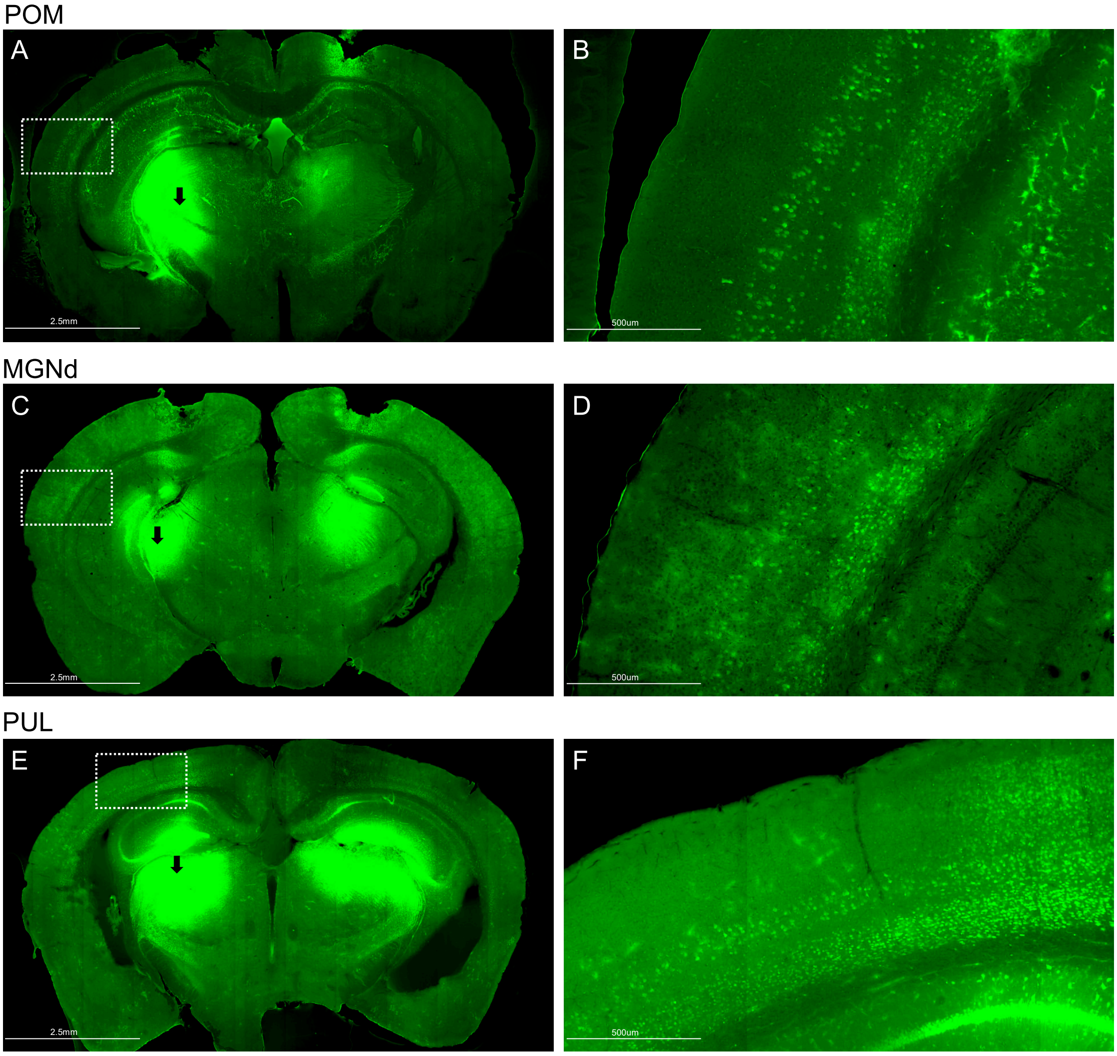

Figure S3. Representative images depicting neuroanatomical tract tracing injections and labeling in CNTNAP2$^{-/-}$ mice of retrograde tracer injected in non-primary sensory thalamic nuclei (A) posterior medial nucleus (POM) (indicated with arrow) with (B) retrogradely labeled cells in S1 (enlarged image indicated by dashed line in A), (C) dorsal division of medial geniculate nucleus (MGNd) (indicated with arrow) with (D) retrogradely labeled cells in A1 (enlarged image indicated by dashed line in C), and (E) lateral posterior thalamic nucleus/pulvinar (PUL) (indicated with arrow) with (F) retrogradely labeled cells in V1 (enlarged image indicated by dashed line in E). Images A and B were horizontally flipped to match orientation with other images. Images A, C and E scale bar 2.5mm. Images B, D and F scale bar 500μm.

Primary Somatosensory Cortex (S1)

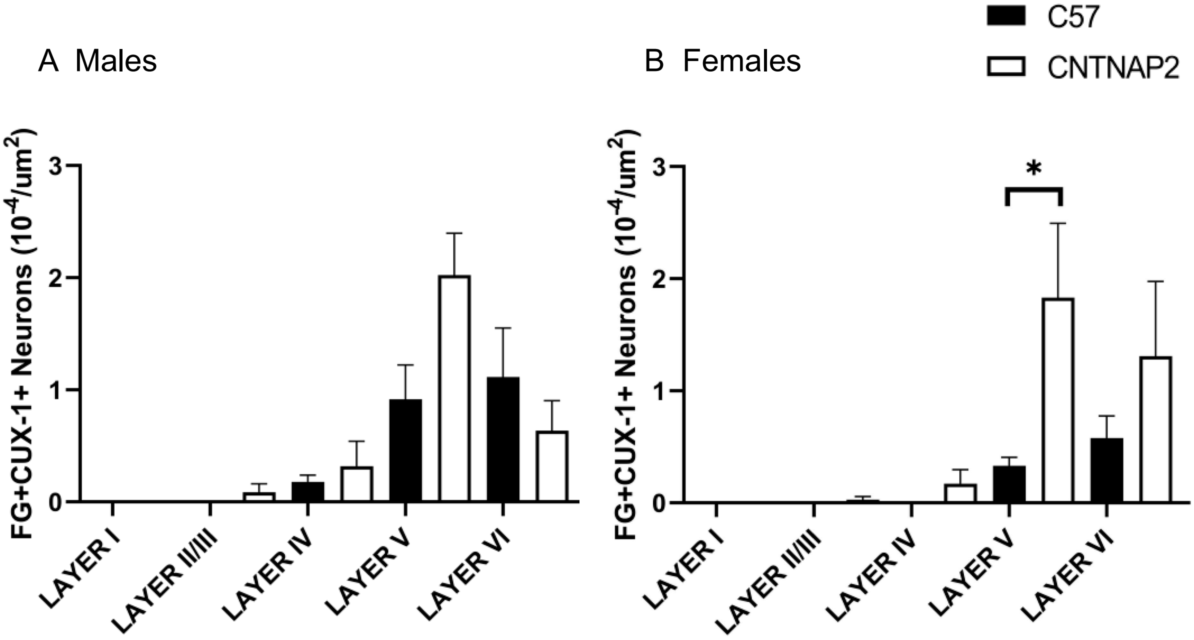

Figure S4. Increased density of double-labeled fluorogold and CUX-1 positive cells in (A) male mice (B) and female mice in S1 region. C57 (n=4 males; n=5 females) and CNTNAP2$^{-/-}$ (n=4 males; n=4 females) mice. Data expressed as mean ± SEM (p<0.05) (p<0.1).

Primary Somatosensory Cortex (S1)

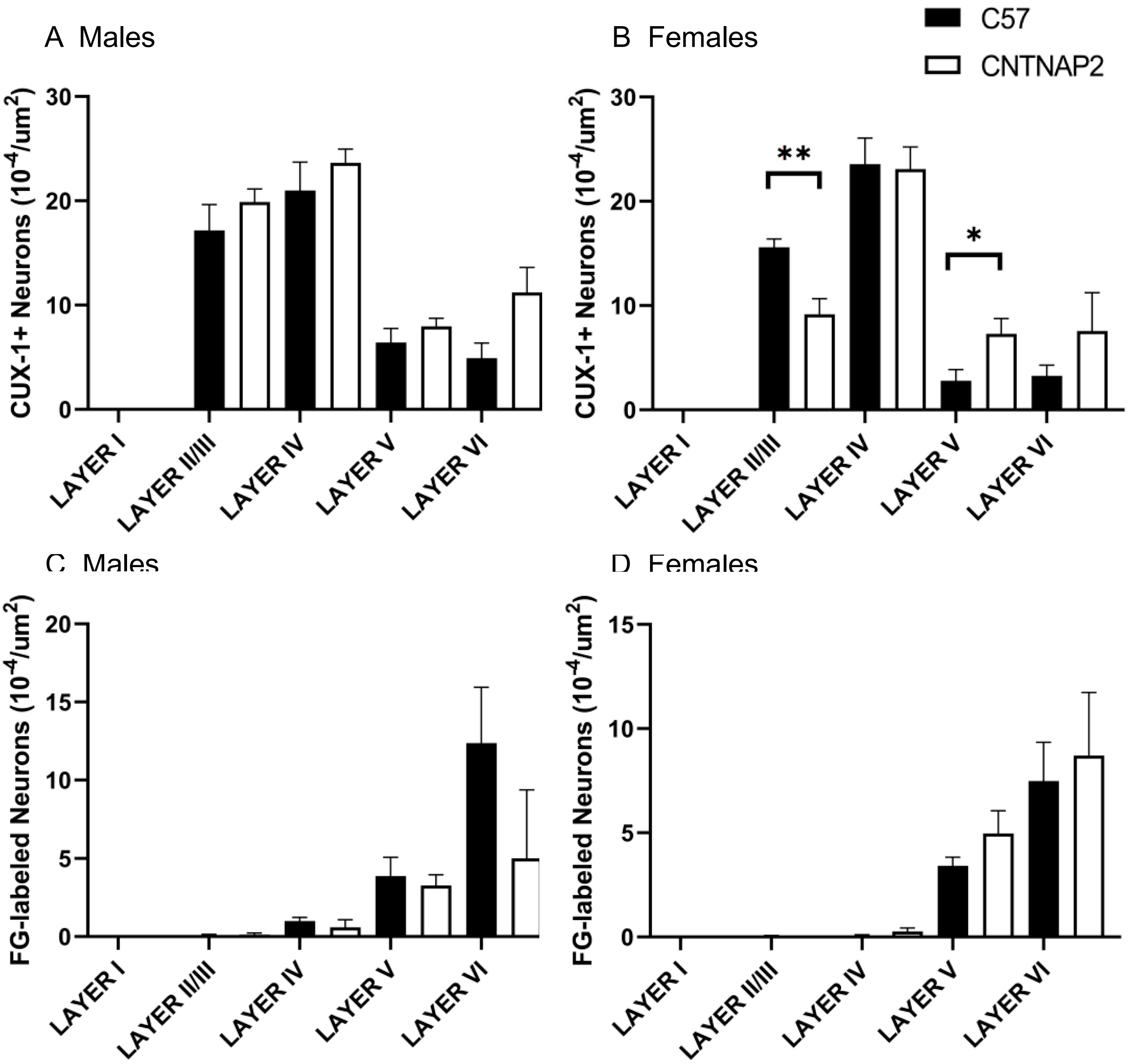

Figure S5. Density of CUX-1 positive cells and fluorogold labeled cells in (A, C) male mice (B, D) and female mice in S1 region. C57 (n=4 males; n=5 females) and CNTNAP2$^{-/-}$ (n=4 males; n=4 females) mice. Data expressed as mean ± SEM (p<0.05) (p<0.1).

Primary Auditory Cortex (A1)

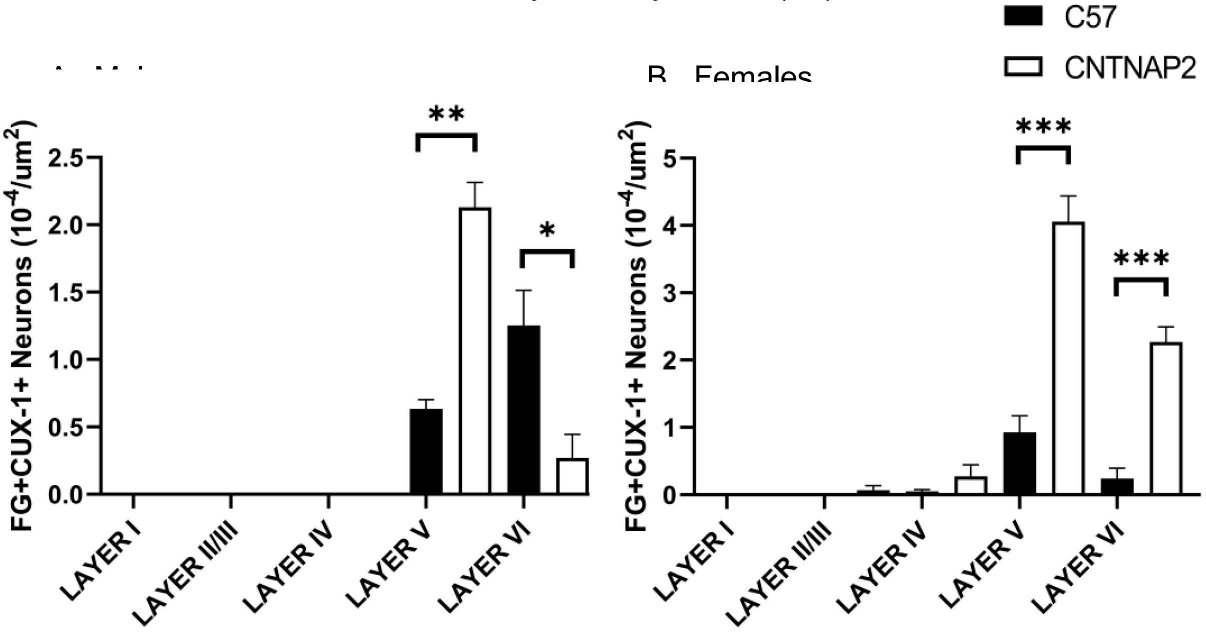

Figure S6. Increased density of double-labeled fluorogold and CUX-1 positive cells in (A) male mice (B) and female mice in A1 region. C57 (n=3 males; n=4 females) and CNTNAP2$^{-/-}$ (n=3 males; n=3 females) mice. Data expressed as mean ± SEM ($p<0.05$).

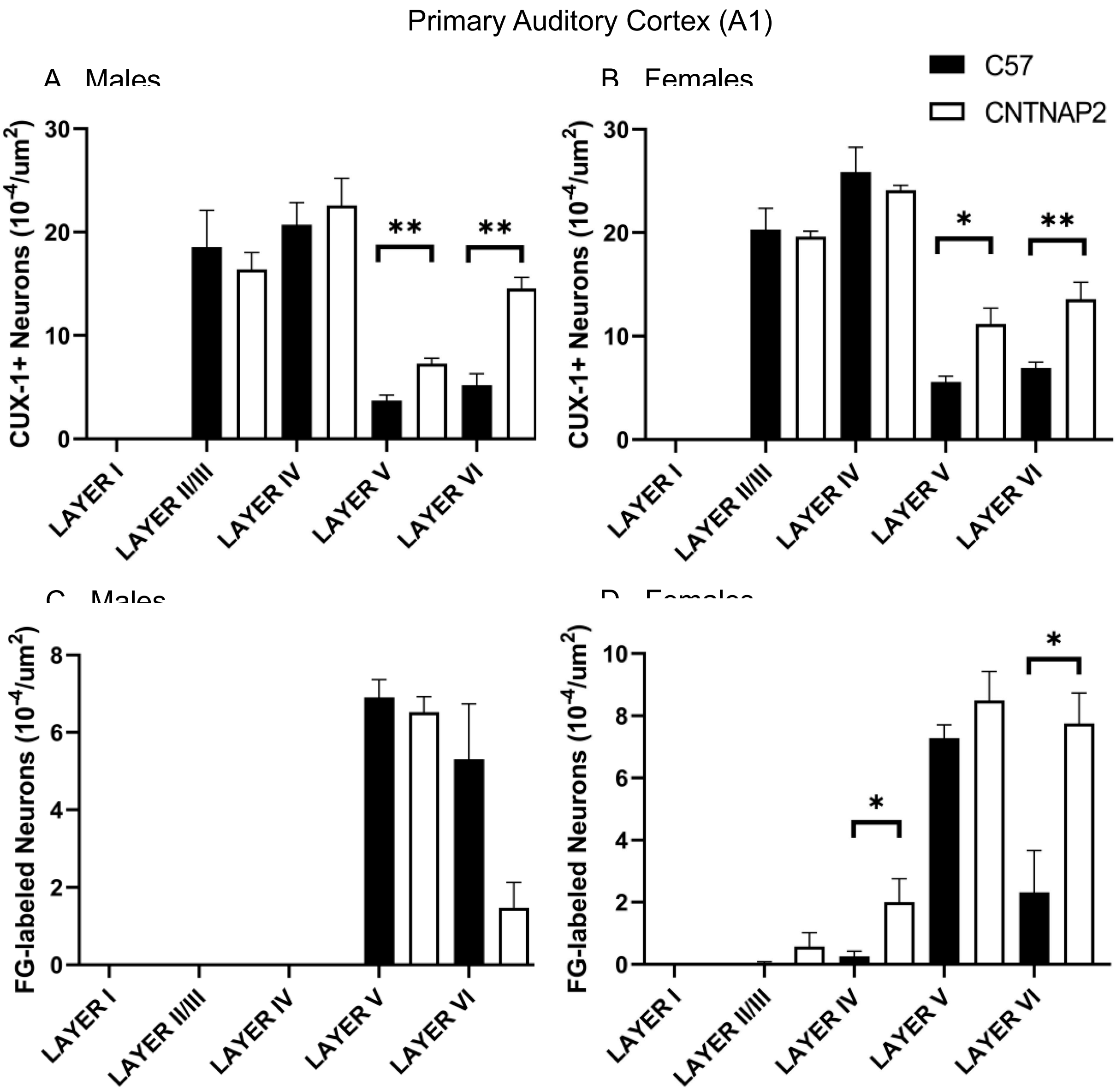

Figure S7. Density of CUX-1 positive cells and fluorogold labeled cells in (A, C) male mice (B, D) and female mice in A1 region. C57 (n=3 males; n=4 females) and CNTNAP2$^{-/-}$ (n=3 males; n=3 females) mice. Data expressed as mean ± SEM (p<0.05) (p<0.1).

Primary Visual Cortex (V1)

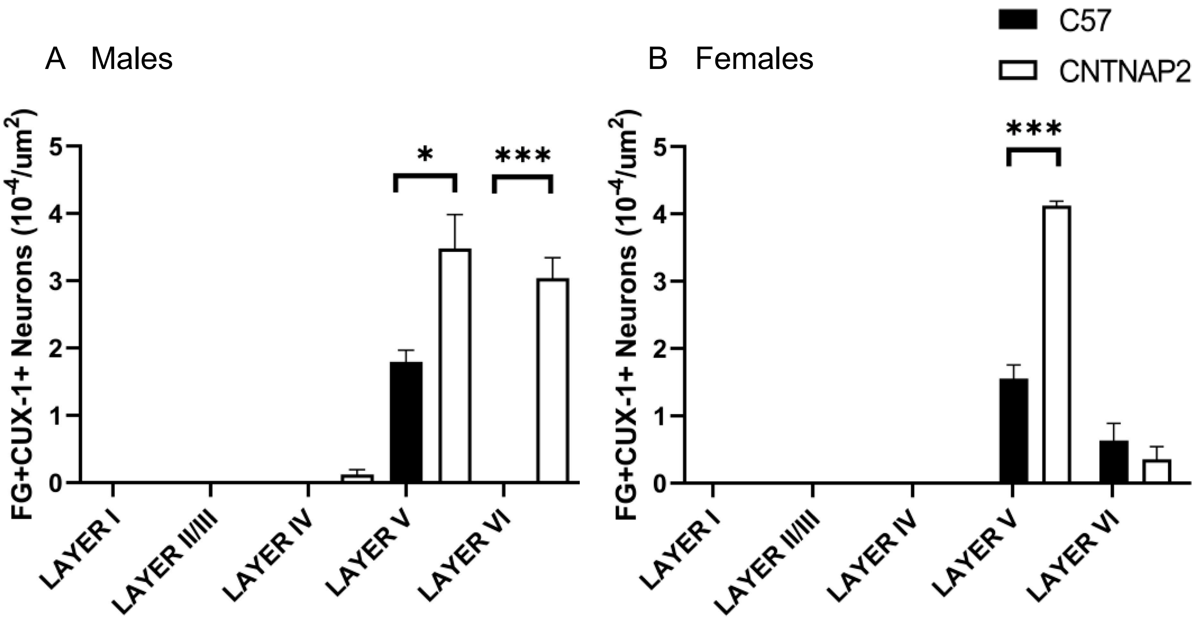

Figure S8. Increased density of double-labeled fluorogold and CUX-1 positive cells in (A) male mice (B) and female mice in V1 region. C57 (n=3 males; n=3 females) and CNTNAP2$^{-/-}$ (n=3 males; n=3 females) mice. Data expressed as mean ± SEM (p<0.05) (p<0.1).

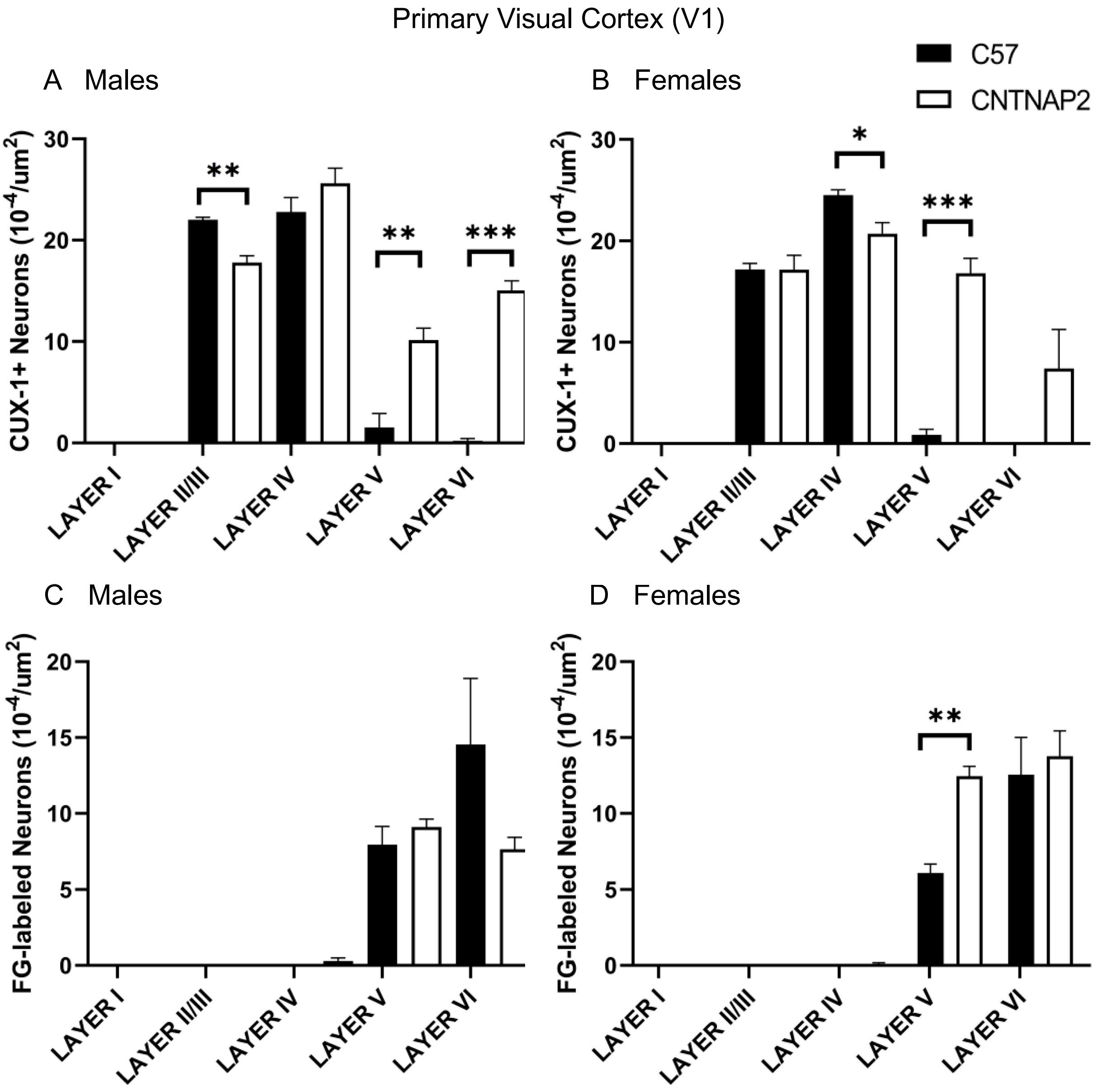

Figure S9. Density of CUX-1 positive cells and fluorogold labeled cells in (A, C) male mice and (B, D) female mice in V1 region. C57 (n=3 males; n=3 females) and CNTNAP2$^{-/-}$ (n=3 males; n=3 females) mice. Data expressed as mean ± SEM (p<0.05) (p<0.1).

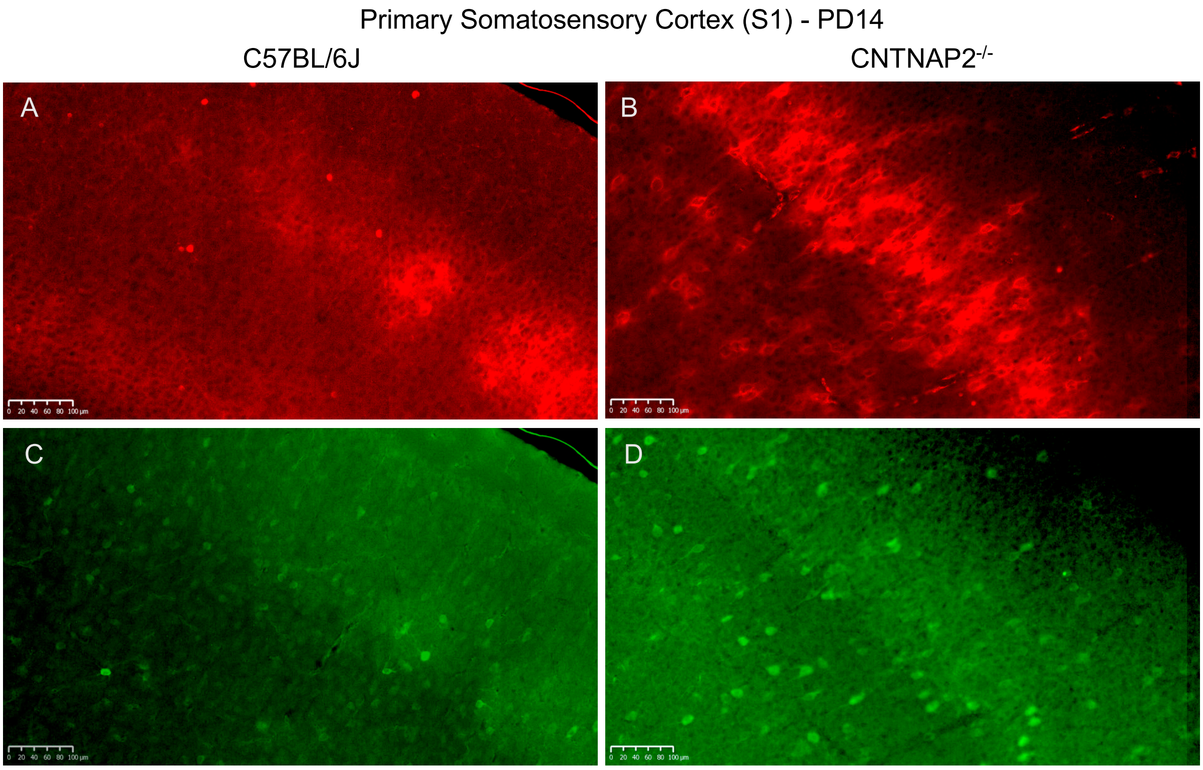

Figure S10. Immunofluorescence images depicting distribution of (A, B) PNNs (red) and (C, D) PV-positive neurons (green) in primary somatosensory cortex (S1) of C57BL/6J and CNTNAP2$^{-/-}$ mice at PD 14. Increase in PNNs and PV neurons in CNTNAP2$^{-/-}$ mice in S1 region. Scale bar 100 μm.

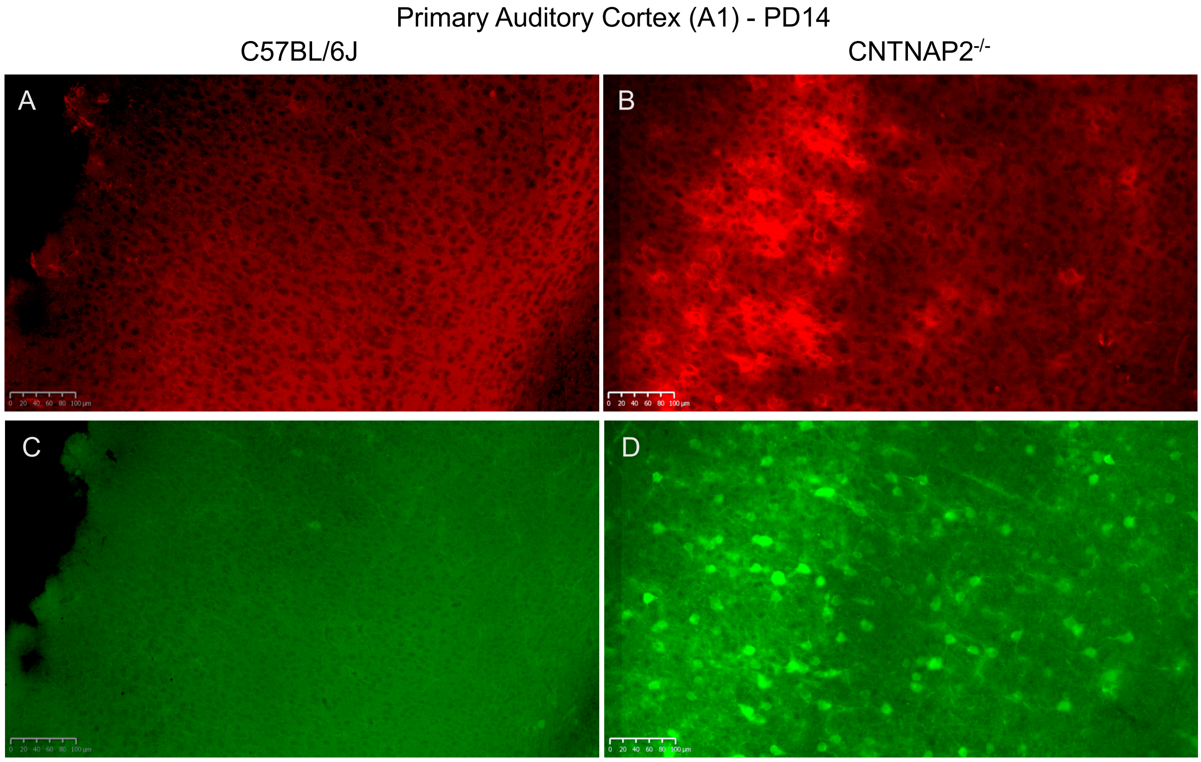

Figure S11. Immunofluorescence images depicting distribution of (A, B) PNNs (red) and (C, D) PV-positive neurons (green) in primary auditory cortex (A1) of C57BL/6J and CNTNAP2$^{-/-}$ mice at PD 14. Increase in PNNs and PV neurons in CNTNAP2$^{-/-}$ mice in A1 region. Scale bar 100 μm.

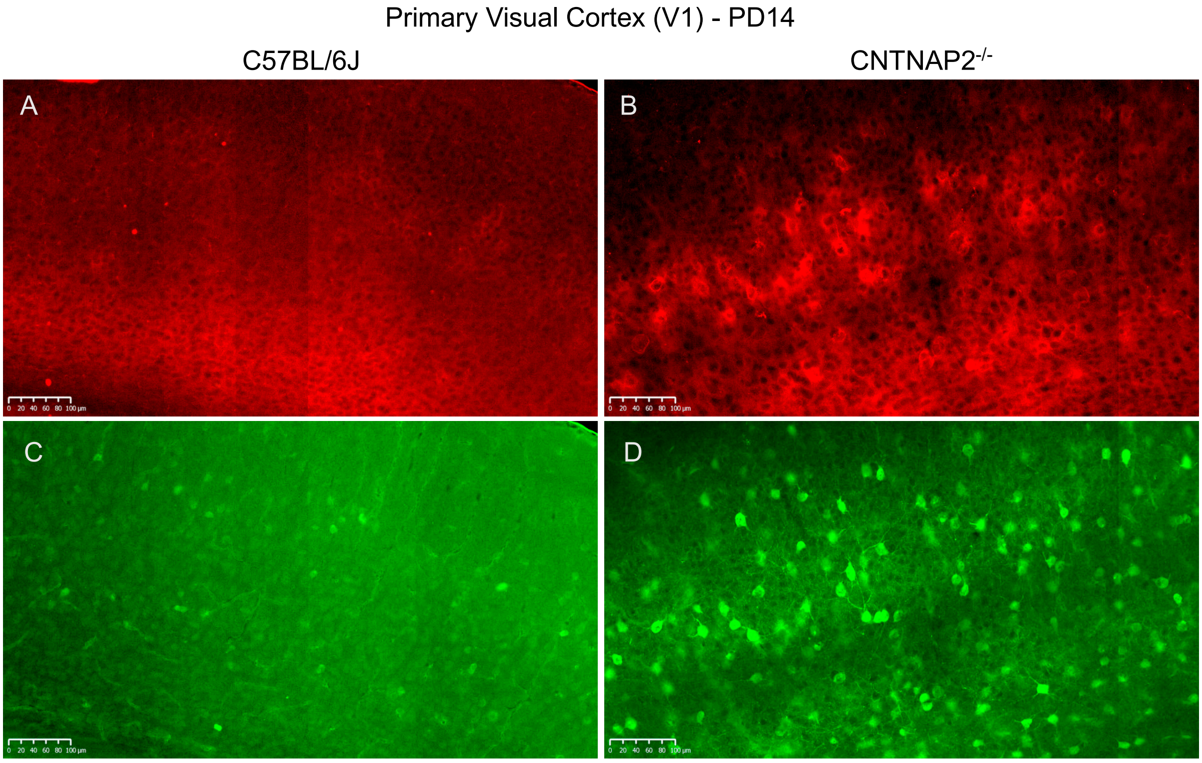

Figure S12. Immunofluorescence images depicting distribution of (A, B) PNNs (red) and (C, D) PV-positive neurons (green) in primary visual cortex (V1) of C57BL/6J and CNTNAP2$^{-/-}$ mice at PD 14. Increase in PNNs and PV neurons in CNTNAP2$^{-/-}$ mice in V1 region. Scale bar 100 µm.

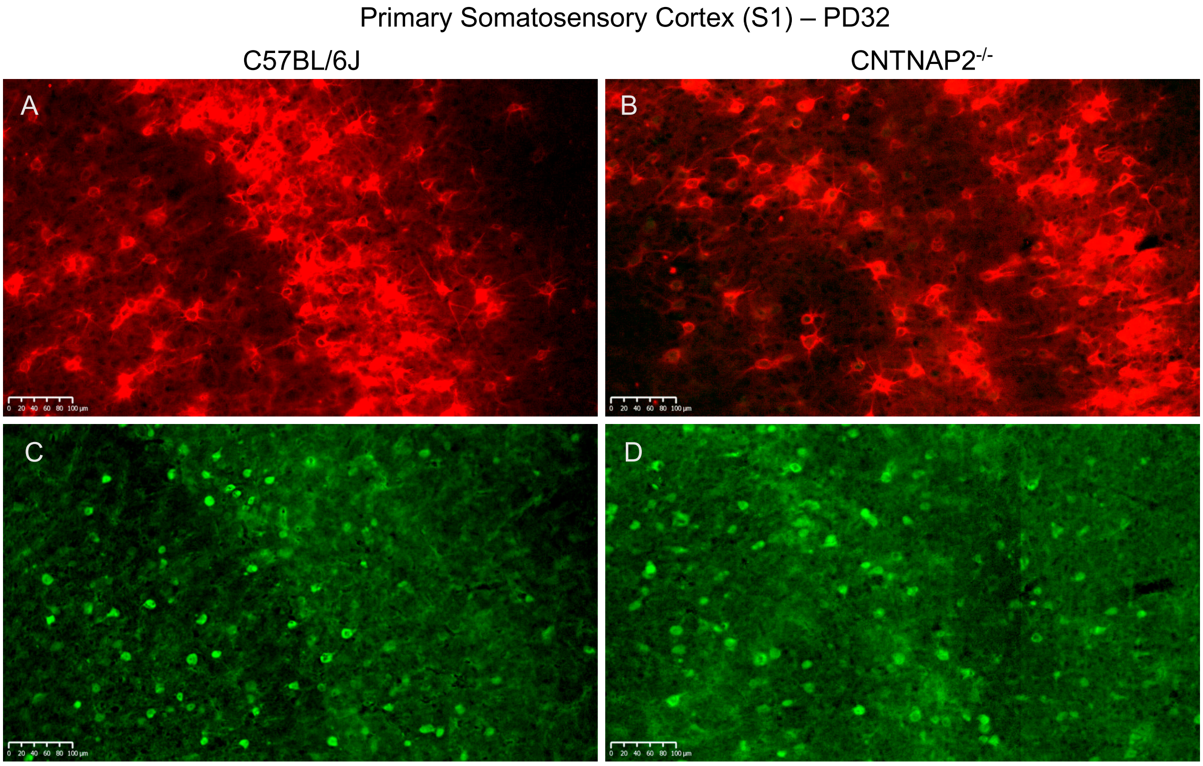

Figure S13. Immunofluorescence images depicting distribution of (A, B) PNNs (red) and (C, D) PV-positive neurons (green) in primary somatosensory cortex (S1) of C57BL/6J and CNTNAP2$^{-/-}$ mice at PD 32. No difference in PNNs and PV neurons in CNTNAP2$^{-/-}$ mice compared to WT mice in S1 region. Scale bar 100 μm.

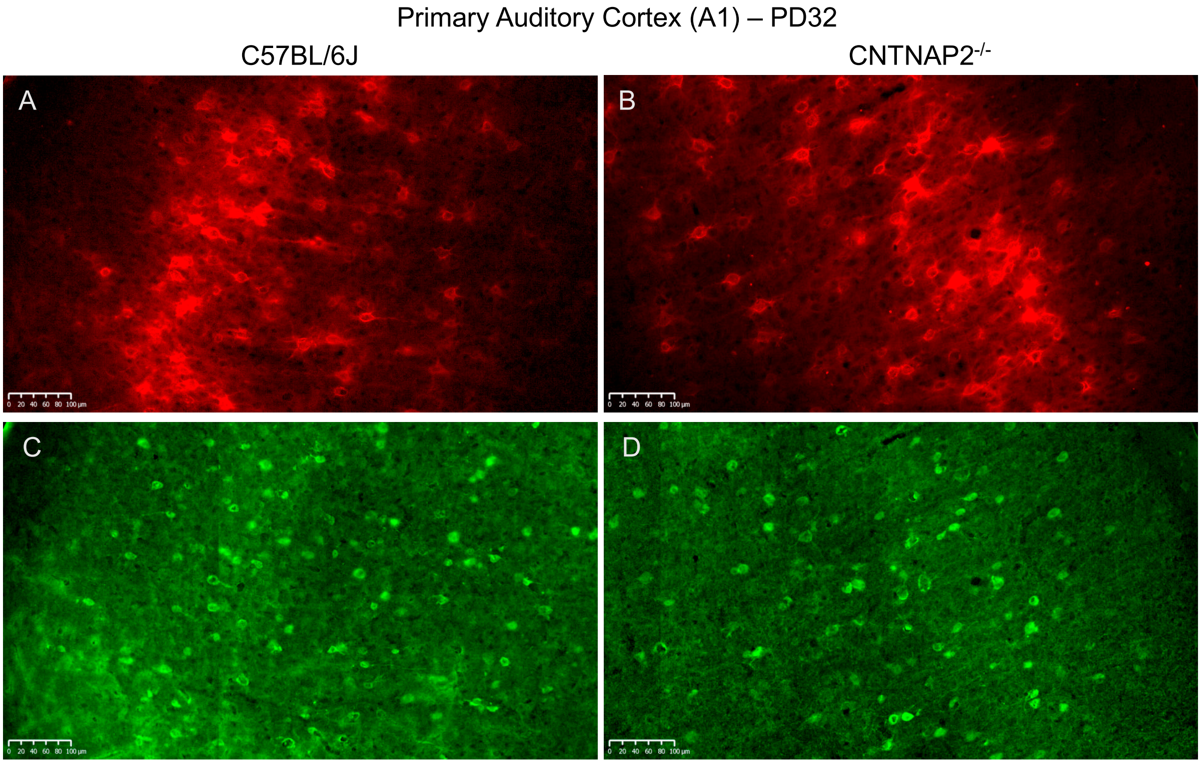

Figure S14. Immunofluorescence images depicting distribution of (A, B) PNNs (red) and (C, D) PV-positive neurons (green) in primary auditory cortex (A1) of C57BL/6J and CNTNAP2$^{-/-}$ mice at PD 32. No difference in PNNs and PV neurons in CNTNAP2$^{-/-}$ mice compared to WT mice in A1 region. Scale bar 100 μm.

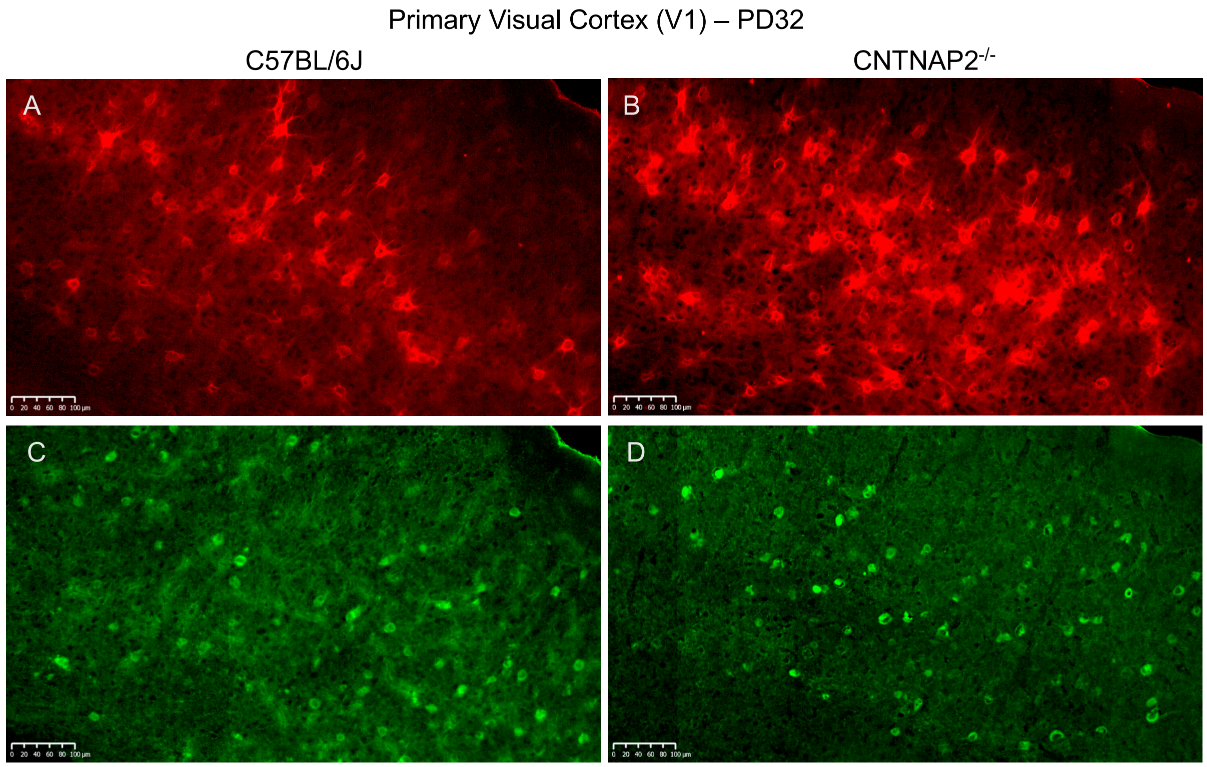

Figure S15. Immunofluorescence images depicting distribution of (A, B) PNNs (red) and (C, D) PV-positive neurons (green) in primary visual cortex (V1) of C57BL/6J and CNTNAP2$^{-/-}$ mice at PD 32. No difference in PNNs and PV neurons in CNTNAP2$^{-/-}$ mice compared to WT mice in V1 region. Scale bar 100 μm.

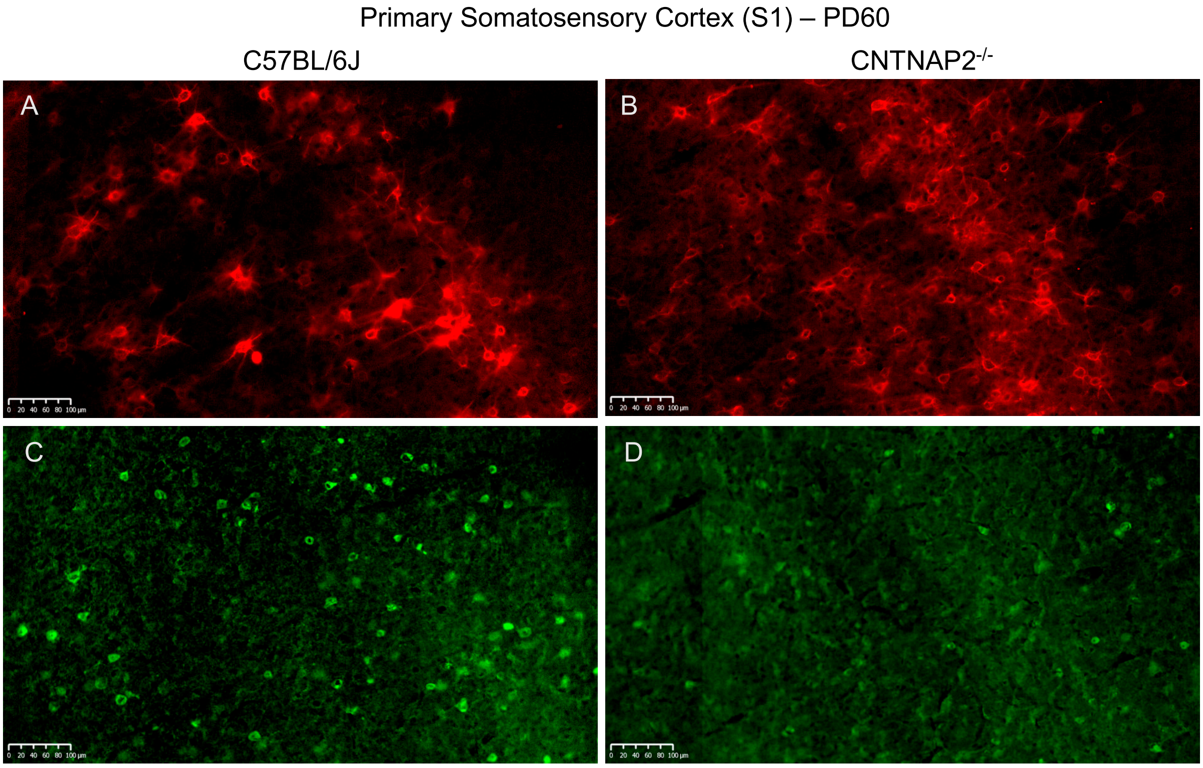

Figure S16. Immunofluorescence images depicting distribution of (A, B) PNNs (red) and (C, D) PV-positive neurons (green) in primary somatosensory cortex (S1) of C57BL/6J and CNTNAP2$^{-/-}$ mice at PD 60. No difference in PNNs and PV neurons in CNTNAP2$^{-/-}$ mice compared to WT mice in S1 region. Scale bar 100 μm.

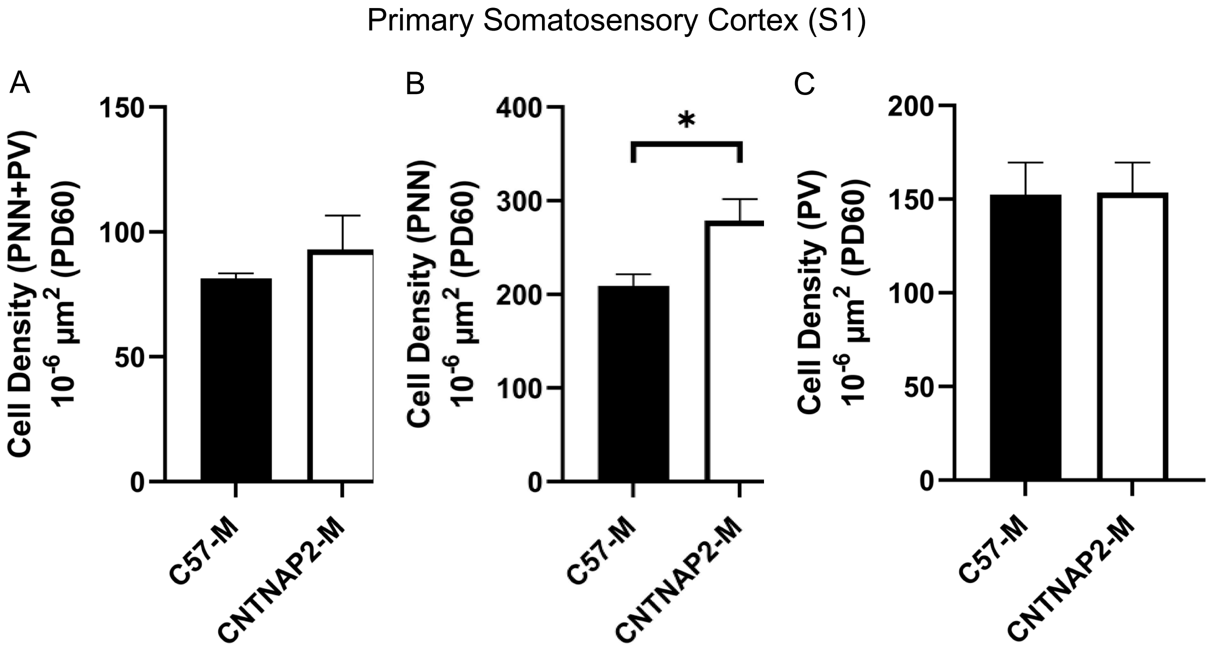

Figure S17. Quantitative estimation of (A) PNNs co-localized with the PV+ neurons, (B) PNNs and (C) PV+ cells in primary somatosensory cortex (S1) of male C57BL/6J (n=4) and CNTNAP2$^{-/-}$ (n=3) mice at PD 60. Significant increase in density of PNNs in male CNTNAP2$^{-/-}$ mice in S1 region. Data expressed as mean ± SEM ($p<0.05$).

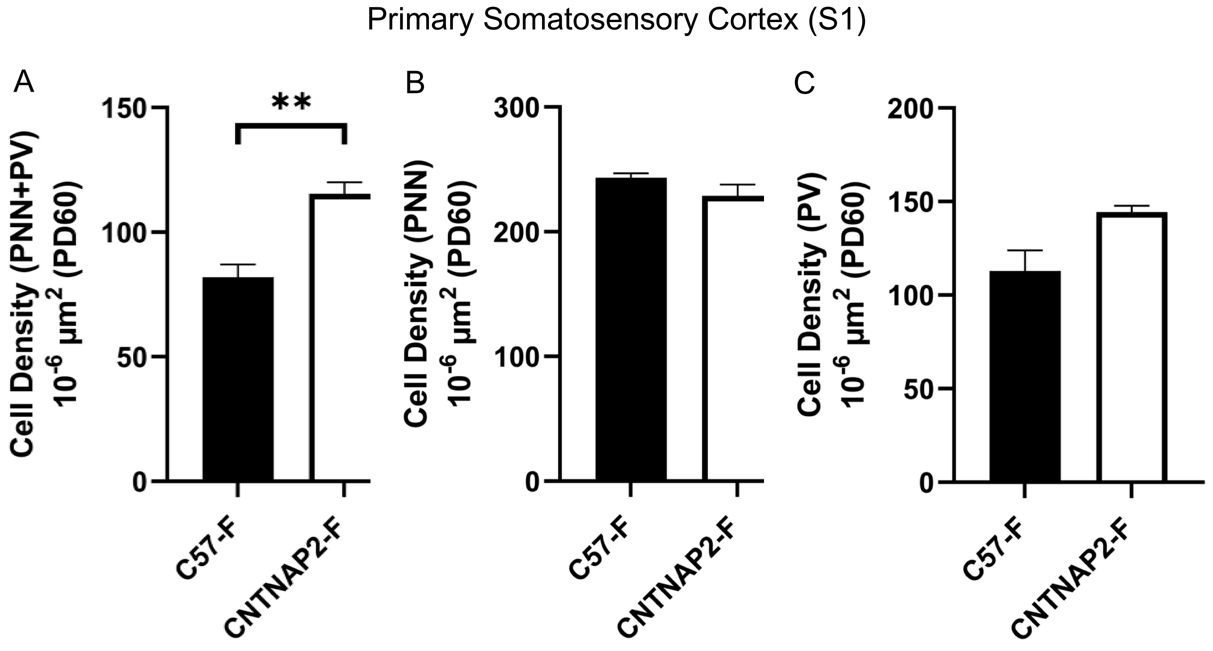

Figure S18. Quantitative estimation of (A) PNNs co-localized with the PV+ neurons, (B) PNNs and (C) PV+ cells in primary somatosensory cortex (S1) of female C57BL/6J (n=4) and CNTNAP2$^{-/-}$ (n=3) mice at PD 60. Significant increase in density of co-localized cells in female CNTNAP2$^{-/-}$ mice in S1 region. Data expressed as mean ± SEM ($p<0.05$) ($p<0.1$).

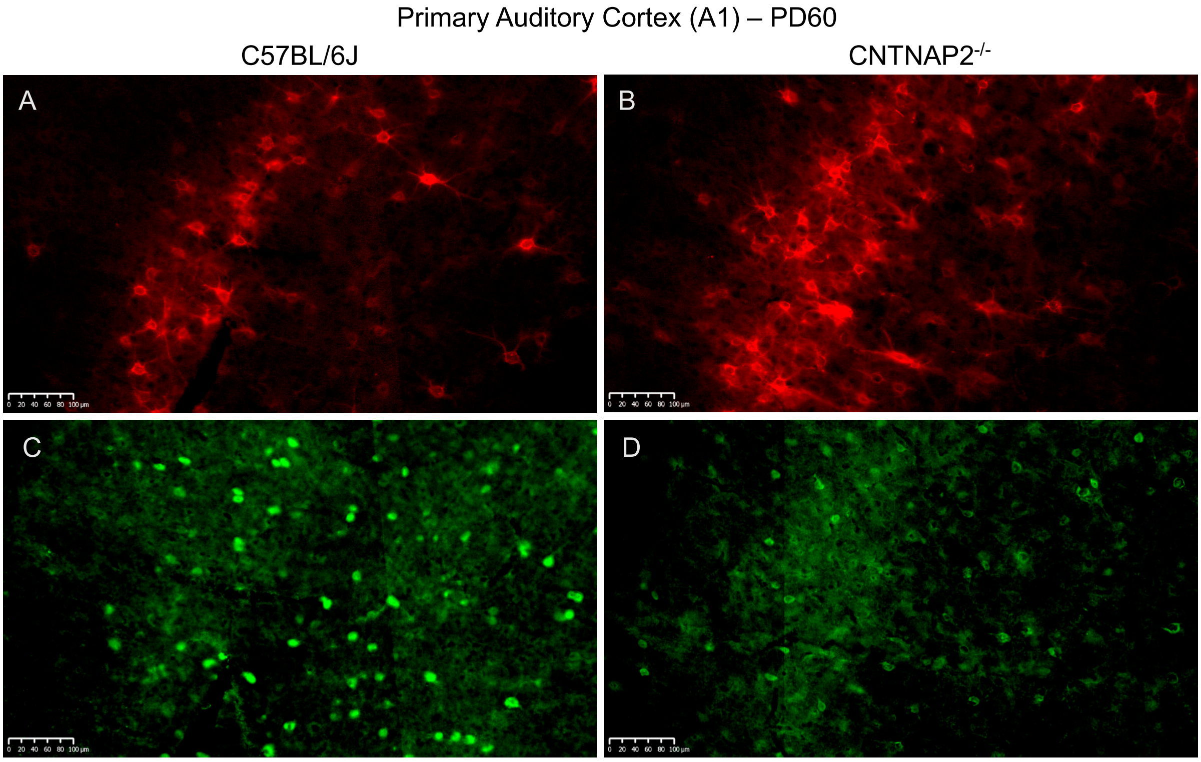

Figure S19. Immunofluorescence images depicting distribution of (A, B) PNNs (red) and (C, D) PV-positive neurons (green) in primary auditory cortex (A1) of C57BL/6J and CNTNAP2$^{-/-}$ mice at PD 60. Increase in PNNs in CNTNAP2$^{-/-}$ mice in A1 region. Scale bar 100 μm.

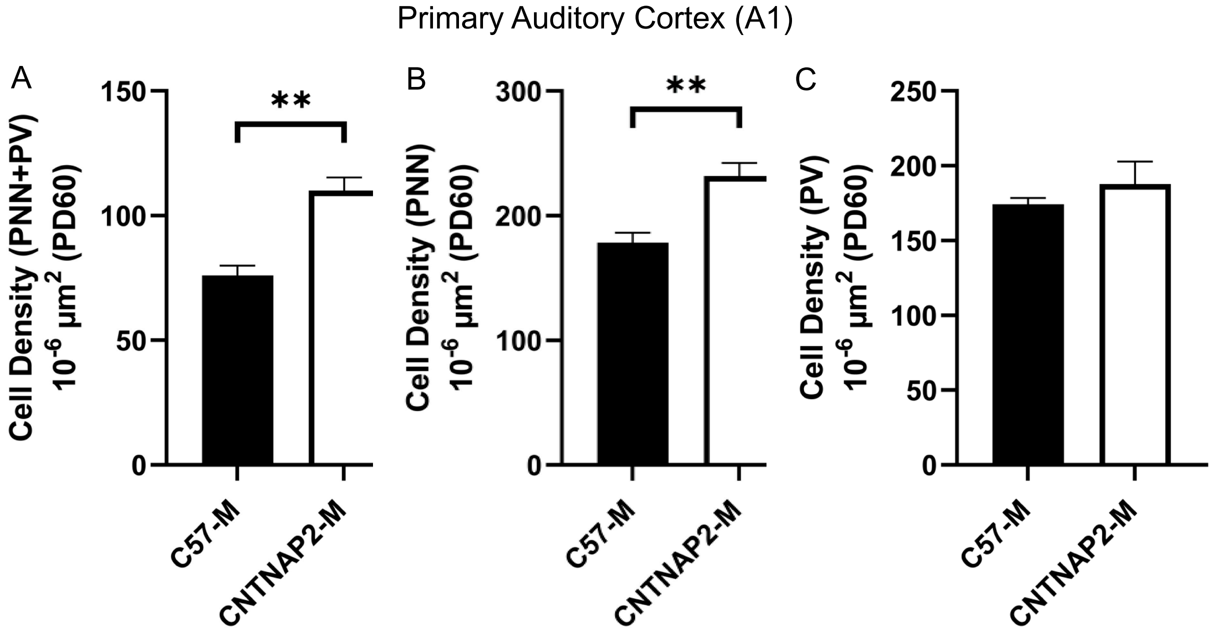

Figure S20. Quantitative estimation of (A) PNNs co-localized with the PV+ neurons, (B) PNNs and (C) PV+ cells in primary auditory cortex (A1) of male C57BL/6J (n=4) and CNTNAP2$^{-/-}$ (n=3) mice at PD 60. Significant increase in density of PNNs and co-localized cells in male CNTNAP2$^{-/-}$ mice in A1 region. Data expressed as mean ± SEM ($p<0.05$).

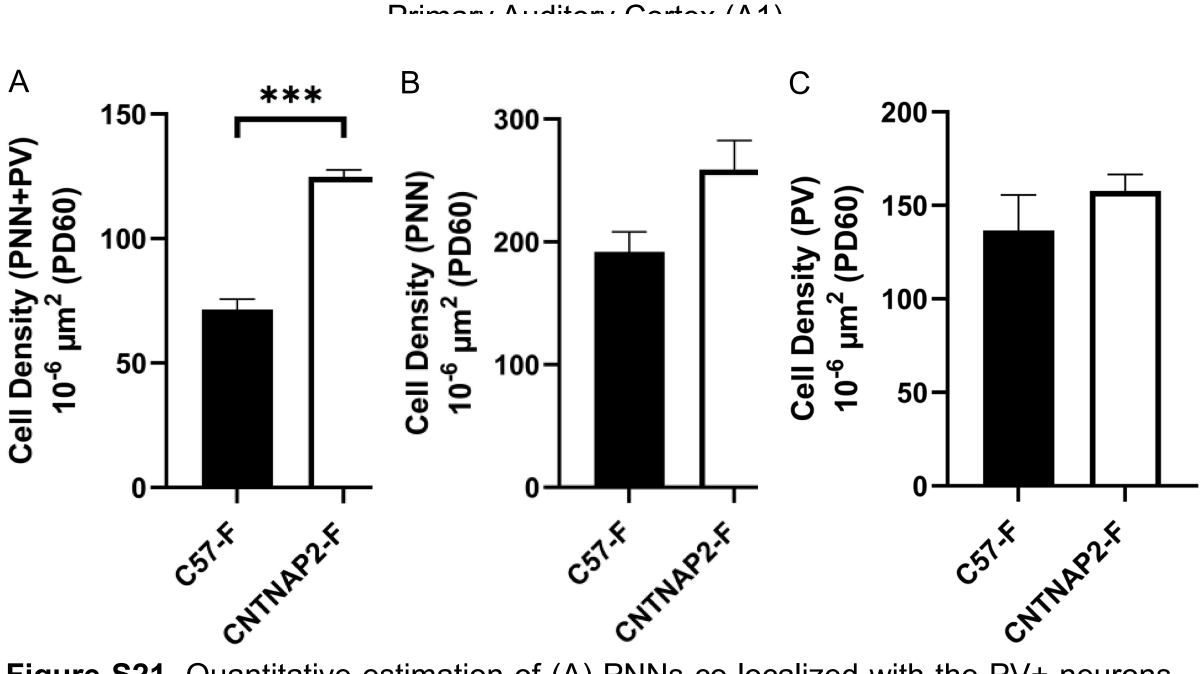

Figure S21. Quantitative estimation of (A) PNNs co-localized with the PV+ neurons, (B) PNNs and (C) PV+ cells in primary auditory cortex (A1) of female C57BL/6J (n=4) and CNTNAP2$^{-/-}$ (n=3) mice at PD 60. Significant increase in density of co-localized cells in female CNTNAP2$^{-/-}$ mice in A1 region. Data expressed as mean ± SEM ($p<0.05$) ($p<0.1$).

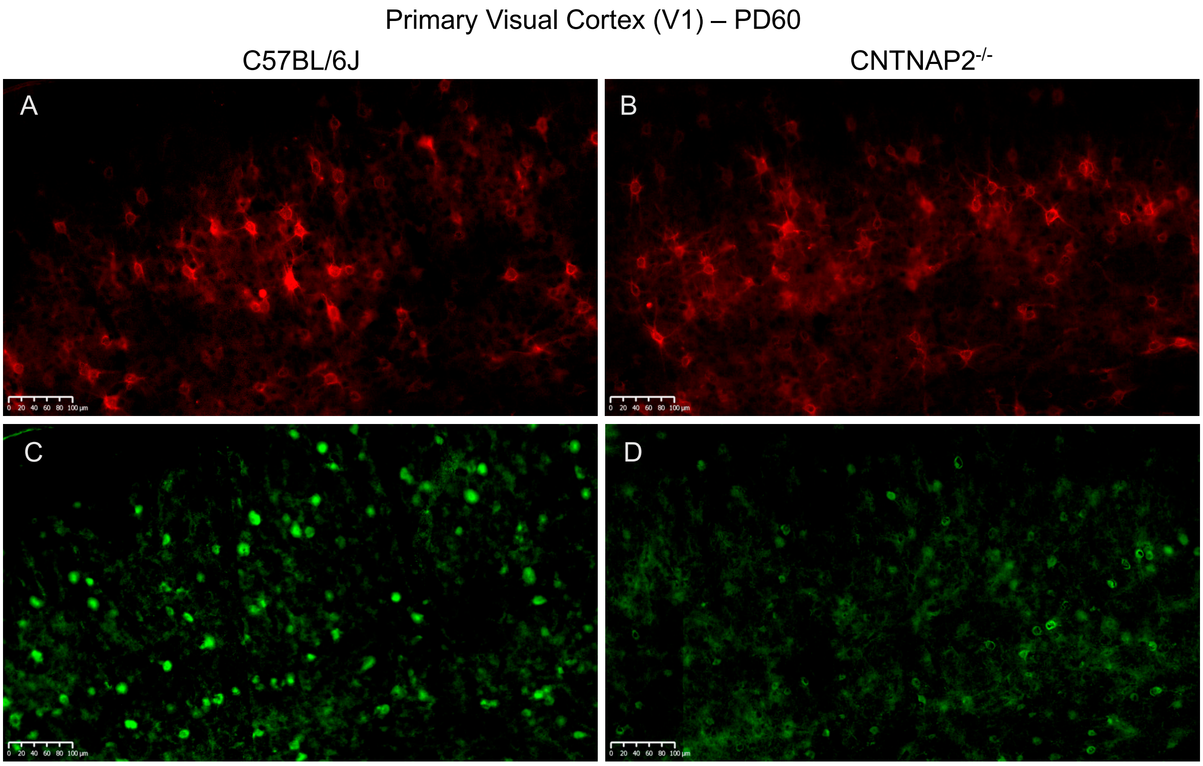

Figure S22. Immunofluorescence images depicting distribution of (A, B) PNNs (red) and (C, D) PV-positive neurons (green) in primary visual cortex (V1) of C57BL/6J and CNTNAP2$^{-/-}$ mice at PD 60. No difference in PNNs and PV neurons in CNTNAP2$^{-/-}$ mice compared to WT mice in V1 region. Scale bar 100 μm.

Primary Visual Cortex (V1)

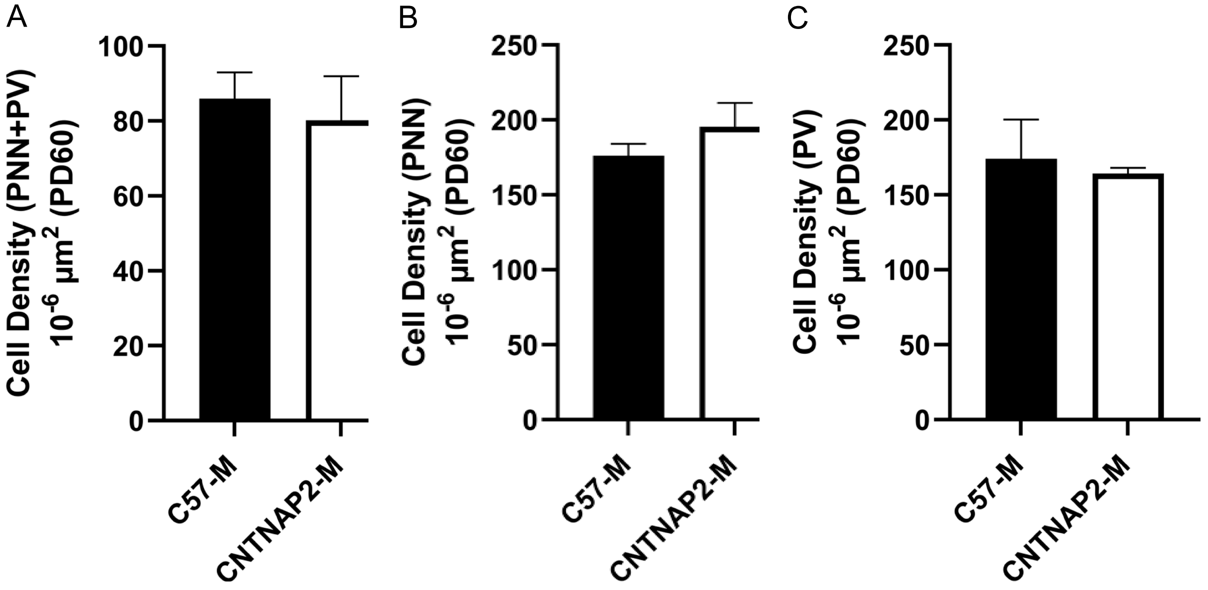

Figure S23. Quantitative estimation of (A) PNNs co-localized with the PV+ neurons, (B) PNNs and (C) PV+ cells in primary visual cortex (V1) of male C57BL/6J (n=4) and CNTNAP2$^{-/-}$ (n=3) mice at PD 60. No difference in density of PNNs, PV neurons and co-localized cells in male CNTNAP2$^{-/-}$ mice compared to WT mice in V1 region.

Primary Visual Cortex (V1)

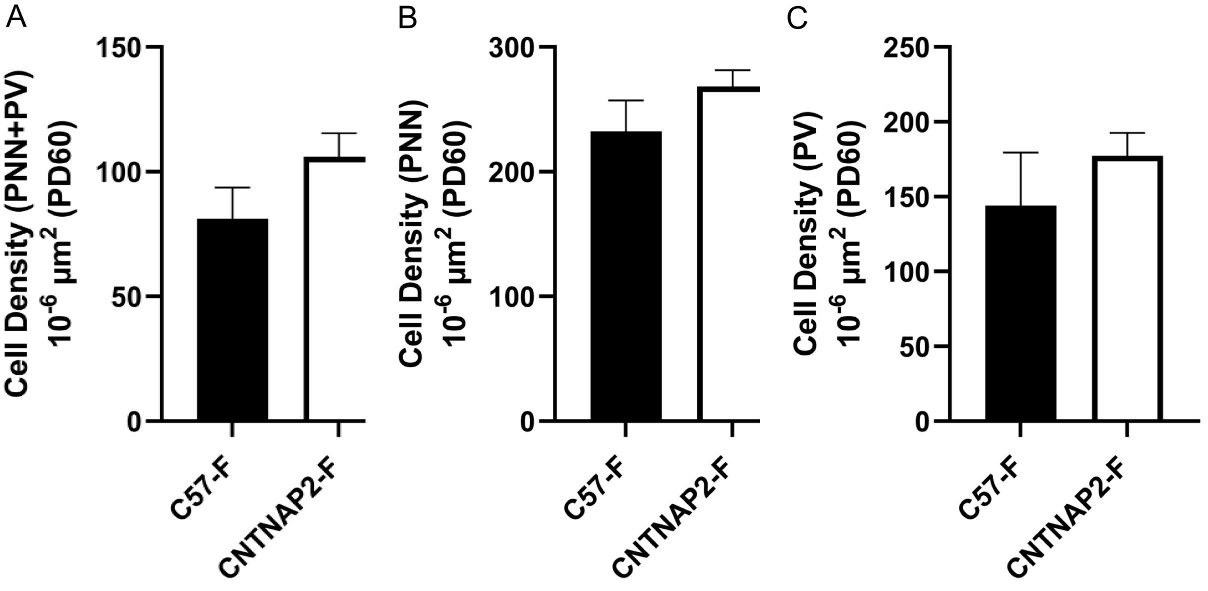

Figure S24. Quantitative estimation of (A) PNNs co-localized with the PV+ neurons, (B) PNNs and (C) PV+ cells in primary visual cortex (V1) of female C57BL/6J (n=4) and CNTNAP2$^{-/-}$ (n=3) mice at PD 60. No difference in density of PNNs, PV neurons and co-localized cells in female CNTNAP2$^{-/-}$ mice compared to WT mice in V1 region.

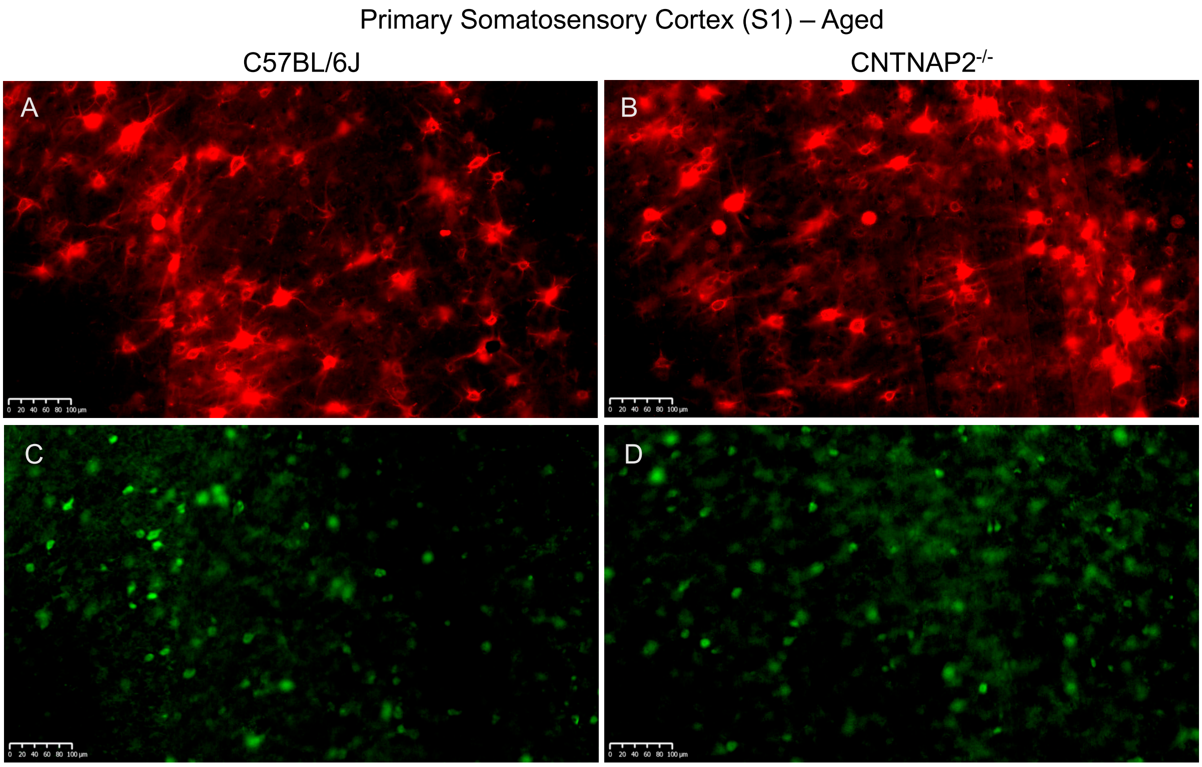

Figure S25. Immunofluorescence images depicting distribution of (A, B) PNNs (red) and (C, D) PV-positive neurons (green) in primary somatosensory cortex (S1) of C57BL/6J and CNTNAP2$^{-/-}$ mice at 13-14 months old (aged). No difference in PNNs and PV neurons in CNTNAP2$^{-/-}$ mice compared to WT mice in S1 region Scale bar 100 μm.

Primary Somatosensory Cortex (S1)

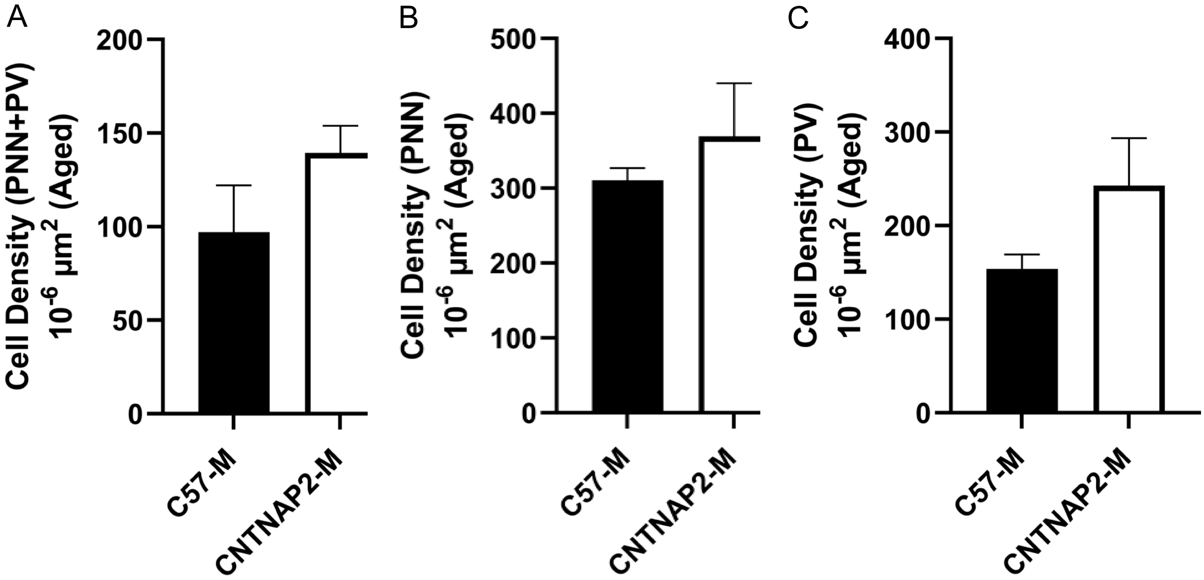

Figure S26. Quantitative estimation of (A) PNNs co-localized with the PV+ neurons, (B) PNNs and (C) PV+ cells in primary somatosensory cortex (S1) of male C57BL/6J (n=4) and CNTNAP2$^{-/-}$ (n=3) mice at 13-14 months old (aged). No difference in density of PNNs, PV neurons and co-localized cells in male CNTNAP2$^{-/-}$ mice compared to WT mice in S1 region.

Primary Somatosensory Cortex (S1)

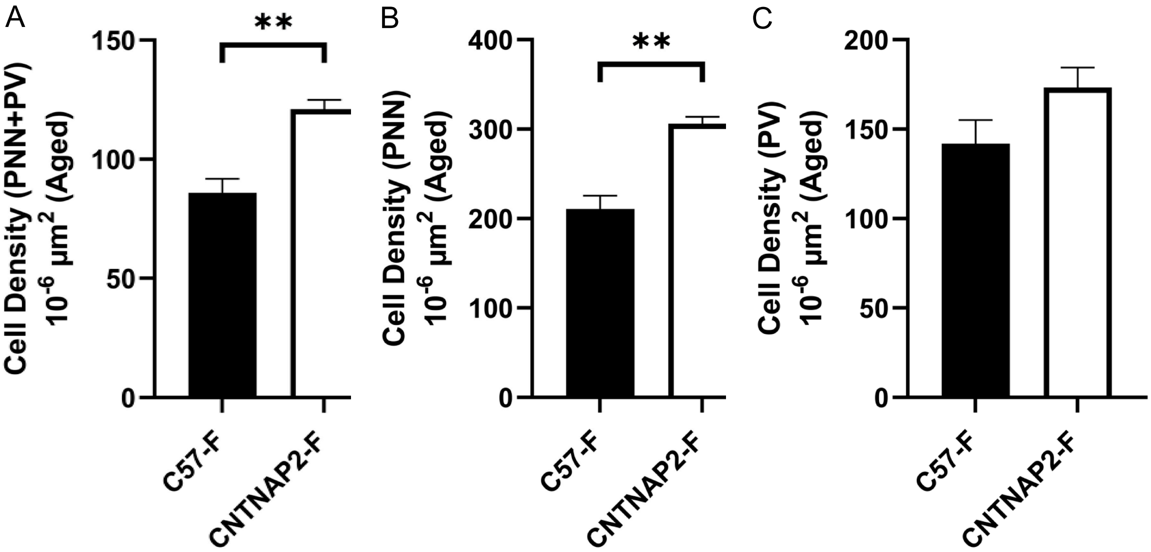

Figure S27. Quantitative estimation of (A) PNNs co-localized with the PV+ neurons, (B) PNNs and (C) PV+ cells in primary somatosensory cortex (S1) of female C57BL/6J (n=3) and CNTNAP2$^{-/-}$ (n=3) mice at 13-14 months old (aged). Significant increase in density of PNNs and co-localized cells in female CNTNAP2$^{-/-}$ mice in S1 region. Data expressed as mean ± SEM ($p<0.05$).

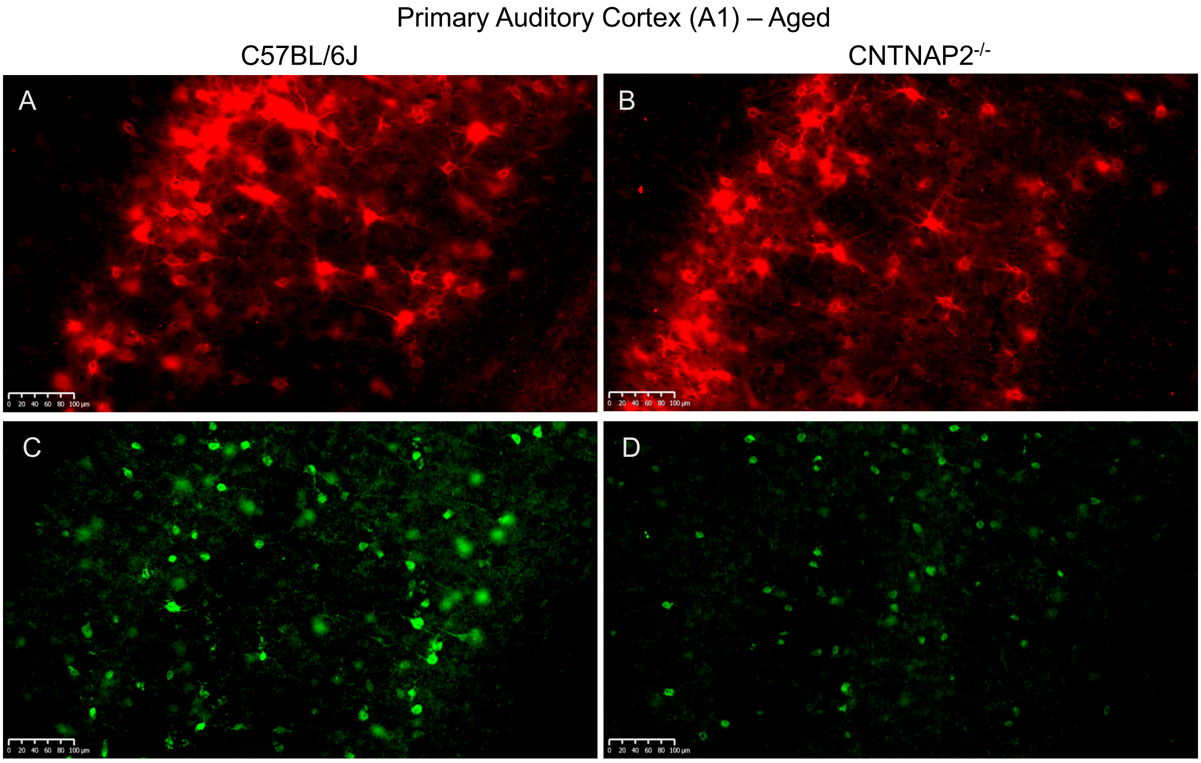

Figure S28. Immunofluorescence images depicting distribution of (A, B) PNNs (red) and (C, D) PV-positive neurons (green) in primary auditory cortex (A1) of C57BL/6J and CNTNAP2$^{-/-}$ mice at 13-14 months old (aged). Increase in PNNs in aged female CNTNAP2$^{-/-}$ mice in A1 region. Scale bar 100 μm.

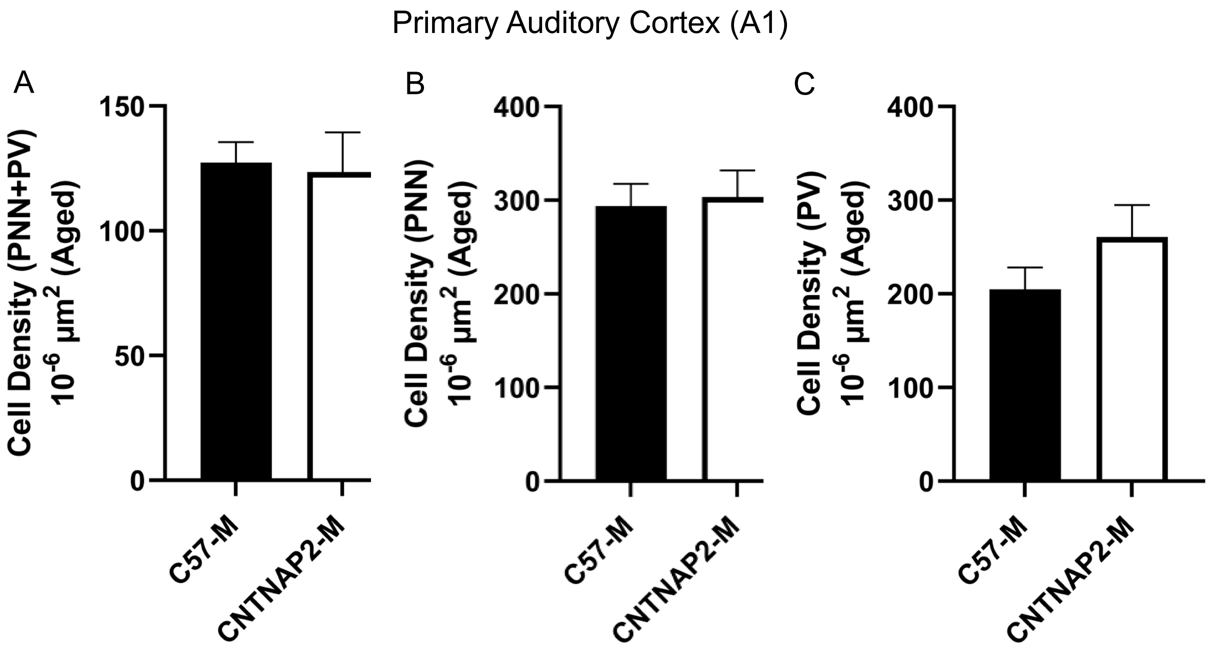

Figure S29. Quantitative estimation of (A) PNNs co-localized with the PV+ neurons, (B) PNNs and (C) PV+ cells in primary auditory cortex (A1) of male C57BL/6J (n=3) and CNTNAP2$^{-/-}$ (n=3) mice at 13-14 months old (aged). No difference in density of PNNs, PV neurons and co-localized cells in male CNTNAP2$^{-/-}$ mice compared to WT mice in A1 region.

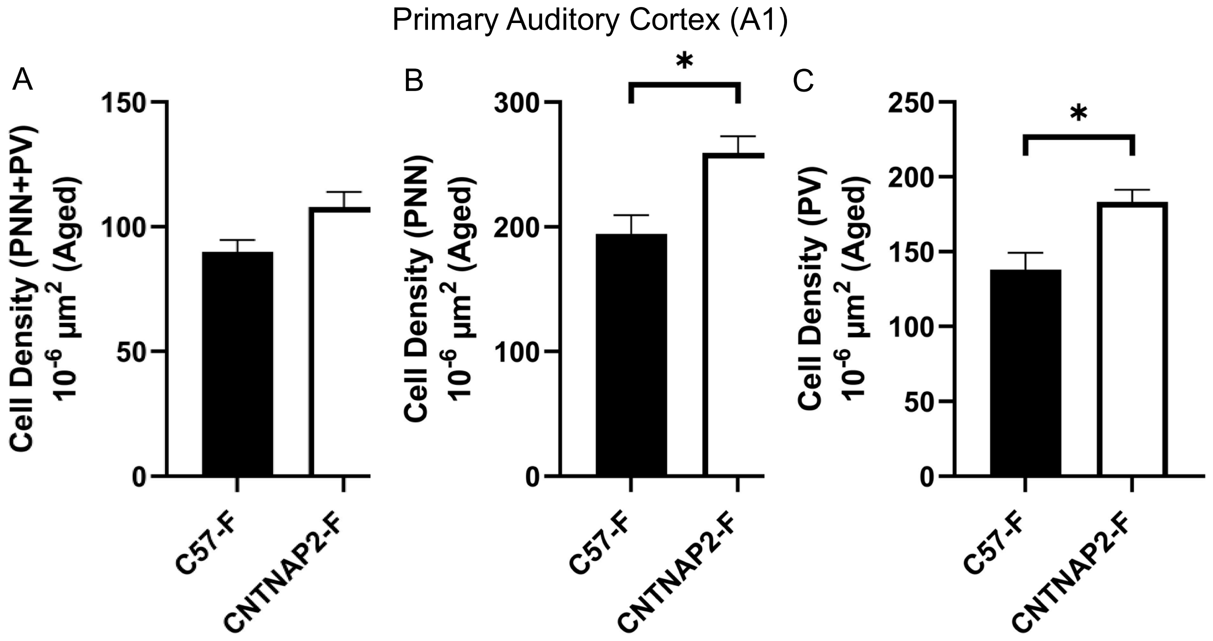

Figure S30. Quantitative estimation of (A) PNNs co-localized with the PV+ neurons, (B) PNNs and (C) PV+ cells in primary auditory cortex (A1) of female C57BL/6J (n=3) and CNTNAP2$^{-/-}$ (n=3) mice at 13-14 months old (aged). Significant increase in density of PNNs and PV cells in female CNTNAP2$^{-/-}$ mice in A1 region. Data expressed as mean ± SEM (p<0.05) (p<0.1).

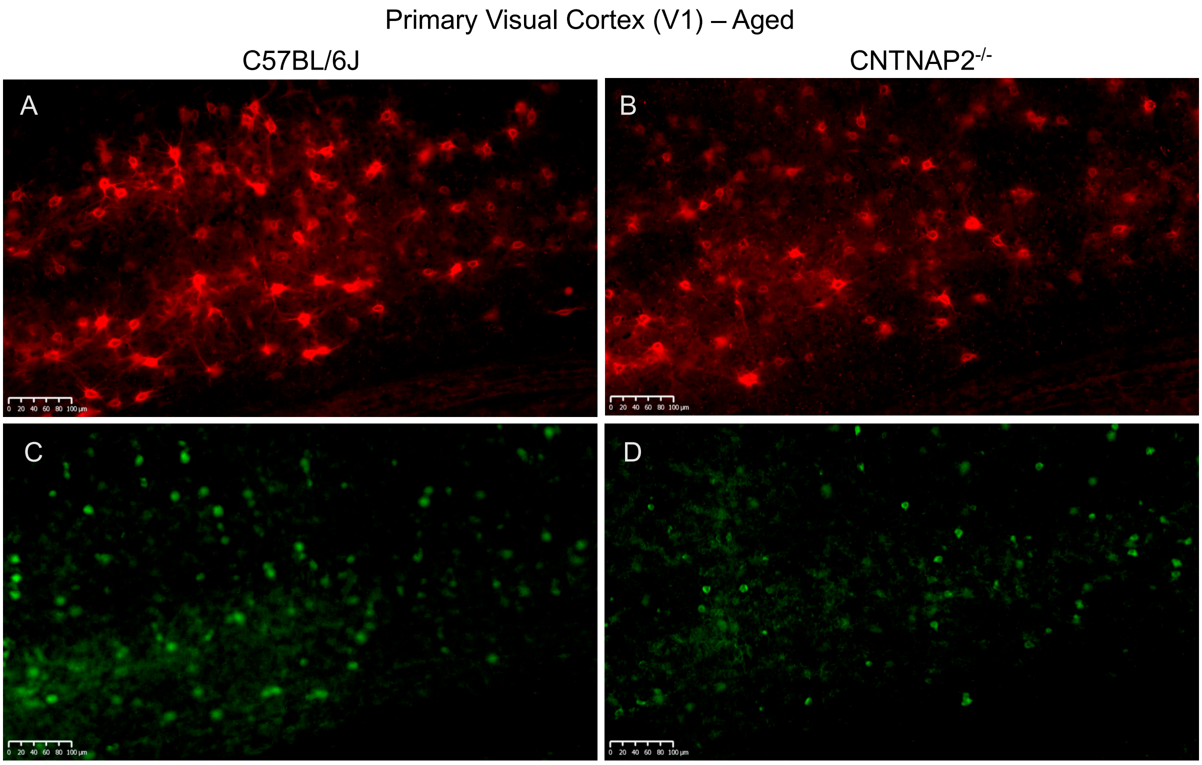

Figure S31. Immunofluorescence images depicting distribution of (A, B) PNNs (red) and (C, D) PV-positive neurons (green) in primary visual cortex (V1) of C57BL/6J and CNTNAP2$^{-/-}$ mice at 13-14 months old (aged). Increase in PNNs in aged female CNTNAP2$^{-/-}$ mice in V1 region. Scale bar 100 μm.

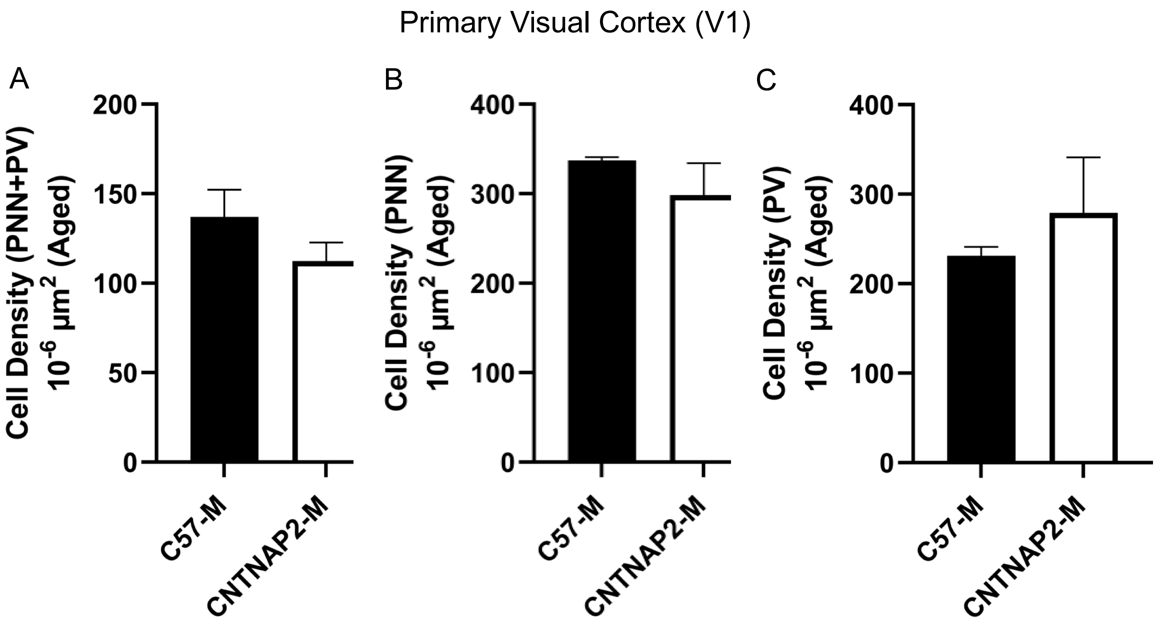

Figure S32. Quantitative estimation of (A) PNNs co-localized with the PV+ neurons, (B) PNNs and (C) PV+ cells in primary auditory cortex (A1) of male C57BL/6J (n=3) and CNTNAP2$^{-/-}$ (n=3) mice at 13-14 months old (aged). No difference in density of PNNs, PV neurons and co-localized cells in male CNTNAP2$^{-/-}$ mice compared to WT mice in V1 region.

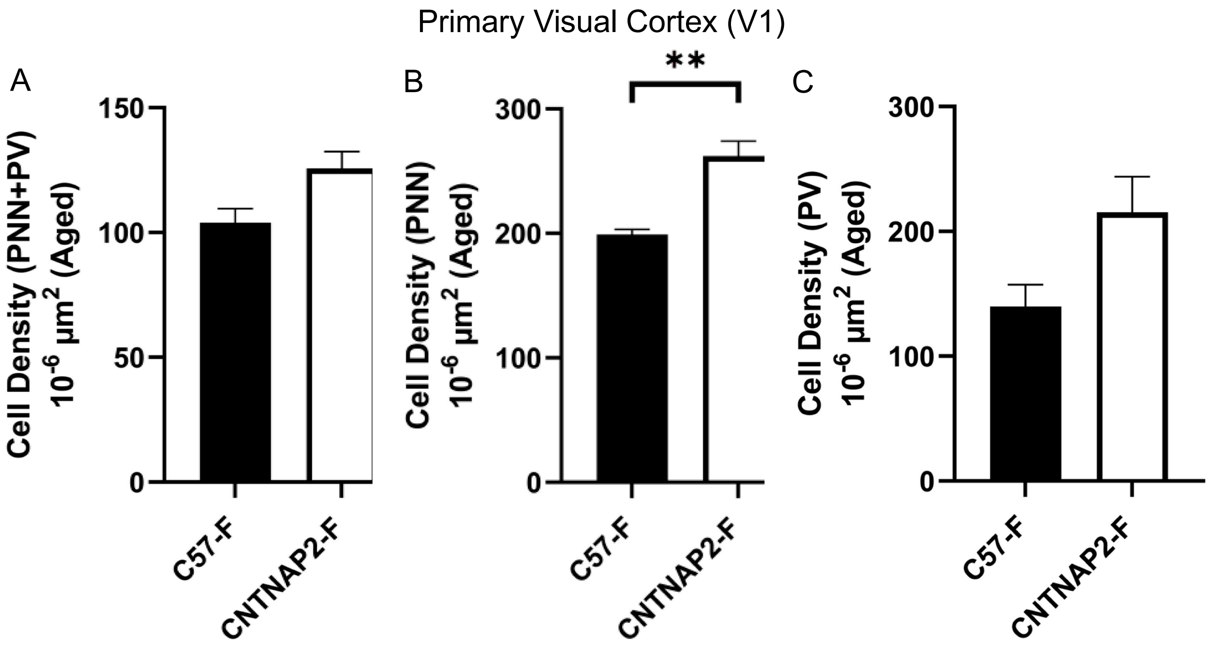

Figure S33. Quantitative estimation of (A) PNNs co-localized with the PV+ neurons, (B) PNNs and (C) PV+ cells in primary auditory cortex (A1) of female C57BL/6J (n=3) and CNTNAP2$^{-/-}$ (n=3) mice at 13-14 months old (aged). Significant increase in density of PNNs in female CNTNAP2$^{-/-}$ mice in V1 region. Data expressed as mean ± SEM ($p<0.05$) ($p<0.1$).
